# Positioning and joining of organic single-crystalline wires

**DOI:** 10.1038/ncomms7737

**Published:** 2015-03-27

**Authors:** Yuchen Wu, Jiangang Feng, Xiangyu Jiang, Zhen Zhang, Xuedong Wang, Bin Su, Lei Jiang

**Affiliations:** 1 Beijing National Laboratory for Molecular Science (BNLMS), Key Laboratory of Organic Solids, Institute of Chemistry, Chinese Academy of Sciences, Beijing 100190, P. R. China; 2 School of Chemistry and Environment, Beihang University, Beijing 100191, P. R. China

## Abstract

Organic single-crystal, one-dimensional materials can effectively carry charges and/or excitons due to their highly ordered molecule packing, minimized defects and eliminated grain boundaries. Controlling the alignment/position of organic single-crystal one-dimensional architectures would allow on-demand photon/electron transport, which is a prerequisite in waveguides and other optoelectronic applications. Here we report a guided physical vapour transport technique to control the growth, alignment and positioning of organic single-crystal wires with the guidance of pillar-structured substrates. Submicrometre-wide, hundreds of micrometres long, highly aligned, organic single-crystal wire arrays are generated. Furthermore, these organic single-crystal wires can be joined within controlled angles by varying the pillar geometries. Owing to the controllable growth of organic single-crystal one-dimensional architectures, we can present proof-of-principle demonstrations utilizing joined wires to allow optical waveguide through small radii of curvature (internal angles of ~90–120°). Our methodology may open a route to control the growth of organic single-crystal one-dimensional materials with potential applications in optoelectronics.

## Abstract (web summary)

Aligned, one-dimensional, single-crystal materials may allow on-demand photon/electron transfer. Here, the authors use a physical vapour transport technique to grow organic single-crystal wires with the guidance of pillar-structured substrates, and perform proof of concept waveguide experiments.

## Graphical abstract

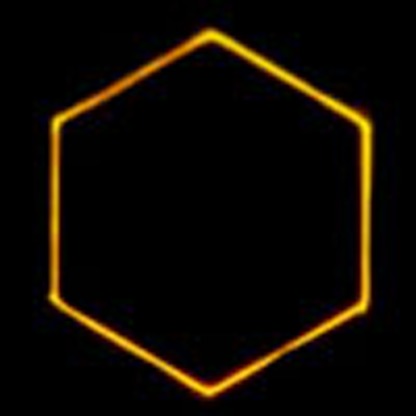

One-dimensional (1D) organic single crystals offer substantial potential for constructing high-efficient optoelectronic systems due to their long-range carrier diffusion and solution-processable on-chip integration^1^,^2^. Various organic semiconductor wires/tubes with ordered molecular stacking and minimized defects has been extensively studied, and fundamental understanding has been gained on material synthesis and structure construction^3^,^4^,^5^,^6^. However, the prepared organic 1D architectures are usually randomly positioned and/or aligned, which restrains the possibility of the on-demand photons/electrons transport^7^,^8^. To control the alignment/position of organic single-crystal 1D architectures, tremendous efforts have been exerted in recent decades^9^,^10^,^11^,^12^,^13^,^14^,^15^,^16^,^17^,^18^,^19^,^20^, and the methods thus developed can be divided into two categories: solution-processing techniques and physical vapour transport (PVT) strategies. Solution-processing techniques employ flow^9^, capillary force^10^,^11^,^12^ or patterned surfaces^13^,^14^,^15^,^16^,^17^,^18^ to drive the self-assembly of micro-/nanostructures in confined regions. Although these assembly methods yield well-aligned arrays on a variety of surfaces, this alignment can be easily disrupted by thermal and dynamic fluctuations. Moreover, it is relatively difficult to control the positions of the assembled 1D structures using such methods. The conventional physical vapour strategy^19^,^20^ employs patterned nanoseeds to position organic single-crystal semiconductors, but the ability to align /position as-prepared 1D structures using this strategy is still an open question. Therefore, controlling both the positioning and alignment of single-crystal organic 1D structures is a significant challenge^21^,^22^,^23^. Recently, a surface-induced epitaxial growth technique^24^, which employs nanosteps on sapphire as a guide for the unidirectional alignment of inorganic GaN nanowires, has provided an alternative method of controlling 1D structures based on surface engineering.

In this work, we show a guided PVT (GPVT) strategy to encourage the growth of organic single-crystal wires with the guidance of pillar-structured substrates. Owing to the surface energy difference between the pillar tops/sidewalls, organic molecules tend to nucleate and grow along the pillar edges, yielding submicrometre-wide, hundreds of micrometres long, highly aligned, single-crystal wire arrays. These organic wire arrays are easy to transfer onto flat polymeric substrates through a simple contact-printing technique. Importantly, these organic single-crystal wires can be joined within controlled angles (for example, ~90–120°) by varying the pillar geometries (for example, square, pentagons or hexagons), indicating a potentially powerful route to rationally design the patterning of organic single-crystal 1D architectures. Furthermore, we show a proof-of-principle demonstration by utilizing joined wires to allow light to waveguide through small radii of curvature (for example, internal angles of ~90–120°). It is expected that this GPVT strategy provides new insights for controlling the growth of organic single-crystal 1D materials with promising optoelectrical applications^21^,^22^,^23^.

## Results

### Aligned single-crystal wire arrays

**Figure 1:**
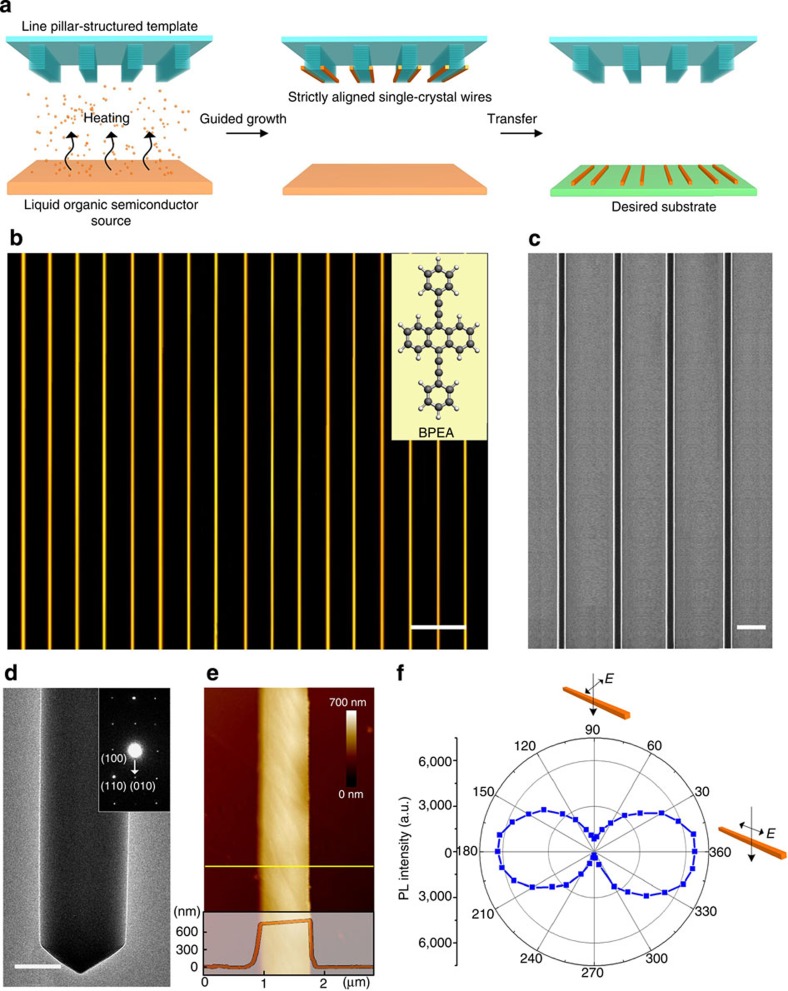
GPVT yields single-crystal wire arrays. (**a**) Schematic illustration of the surface-induced growth of organic, 1D structures. A grooved silicon substrate can encourage preferential crystal nucleation and growth of organic semiconductors in a PVT system. As a result, aligned organic wires become anchored to the tops of the pillar sidewalls. The highly aligned organic wire arrays can be easily transferred onto a flat, polymeric film through physical pressing. (**b**) A dark-field fluorescent micrograph excited using a 325-nm ultraviolet irradiation and (**c**) a scanning electron microscope image of highly aligned BPEA wires on a flat PDMS film. The inset image in **b** depicts the molecular structure of BPEA. (**d**) A TEM image of a single BPEA wire. The inset image presents the corresponding SAED pattern, indicating that the wire had a single-crystal structure and grew along the [010] direction of the BPEA crystal. (**e**) Atomic force microscopy observation of a single BPEA wire. The top surface of this 1D structure was smooth and exhibited only a few steps, indicating a single crystal structure. (**f**) Angle-dependent polarized investigation confirmed the single-crystalline nature of the BPEA wires prepared using the GPVT strategy. The maximum and minimum PL intensity values were observed parallel (180° and 360°) and vertical (90° and 270°) to the direction of the electric field, respectively. Scale bars, **b** 20 μm, **c** 5 μm, **d** 400 nm.

The GPVT system contains two temperature zones: the organic semiconductors sublimate in the high-temperature zone and are crystallized on a substrate in the low-temperature zone^2^. The difference between our method and the conventional PVT technique is that we utilize a pillar-structured substrate to guide the crystal nucleation and the growth of organic single crystals (see Fig. 1a). In the conventional process, the organic semiconductors nucleation sites are randomly distributed on a flat substrate, yielding irregular structures after the crystal-growth process^19^,^20^. By contrast, pillar-structured surfaces have been demonstrated to effectively guide liquid the flow and the growth of 1D structures in our previous reports^25^. In this work, we employed a pillar-structured substrate to guide the vapour flow, thereby controlling the crystal growth of organic semiconductors. A grooved silicon substrate with a width of 10 μm, separation of 10 μm and depth of 20 μm (Supplementary Fig. 1) has been placed in the low-temperature zone to direct the flow of the organic vapour. 9,10-bis(phenylethynyl)anthracene (BPEA) has been selected to demonstrate this GPVT strategy because of its good solubility, thermal stability and, especially, its remarkably high emission efficiency (with a solid-state fluorescence quantum yield of 89.6±0.3%; refs 26, 27, 28) and was placed in the high-temperature zone.

With the guidance of the surface microstructures, the as-prepared BPEA wires become anchored to the tops of each pillar sidewall (Supplementary Fig. 2), yielding highly aligned organic wire arrays, which can be easily transferred onto a flat polymer substrate. Strictly aligned organic wire arrays can be generated by applying a suitable heating temperature and growth time during the GPVT process (see the Methods). To investigate the morphology of the wire array, we acquired dark-field fluorescent micrographs (under excitation by ultraviolet irradiation at 325 nm, see Fig. 1b) and scanning electron microscope image (Fig. 1c) of aligned BPEA wires that had been transferred to a flat polydimethylsiloxane (PDMS) film. In contrast to the random positioning or discontinuous organic wires that have been reported in previous studies^9^,^10^,^11^,^12^,^13^,^14^,^15^,^16^,^17^,^18^,^19^,^20^, each organic 1D structure was found to be uniform (963±46 nm in width and 792±61 nm in height after growth for 20 min, Supplementary Fig. 3), continuous (hundreds of micrometres in length), precisely positioned (the gap between adjacent wires was 10 μm, consistent with the pillar geometry) and highly aligned (tilting angle <1°). Because of the close-packed aggregation of the BPEA molecules along these regular 1D structures, their photoluminescence (PL) spectrum (Supplementary Fig. 4) exhibited a considerable red-shift by as much as 80 nm with respect to the spectrum of the monomers, and the emission colour changed from green to yellow. In the control experiment, a flat substrate, instead of the grooved substrate, was used to repeat the same PVT process. Only randomly positioned and aligned BPEA wires were obtained (Supplementary Fig. 5), demonstrating the importance of the surface microstructures in the alignment of the organic 1D structures. Furthermore, we showed that these aligned BPEA wire arrays could be prepared in a nitrogen environment as well as at atmosphere (Supplementary Fig. 7, 8 and Supplementary Note 1).

The crystal structures of the as-prepared organic 1D structures were carefully investigated. A typical transmission electron microscope (TEM) image of a single BPEA wire (Fig. 1d) shows a smooth, faceted surface from the middle to the edges. The selected-area electron diffraction (SAED) pattern reveals that the organic wire had a single-crystal structure and grew along the [010] direction of the BPEA crystal (inset image in Fig. 1d). As shown in the atomic force microscopy image presented in Fig. 1e, the top surfaces of these BPEA wires are smooth and exhibit only a few steps, reflecting the high crystallinity of the as-prepared 1D structures. In addition to direct TEM/atomic force microscopy observation, cross-polarized micrographs also confirmed the single-crystalline nature of the BPEA wire arrays (Supplementary Fig. 6). The appearances of the ordered organic wires changed from bright to dark simultaneously along their entire length when these linear structures were rotated about an axis perpendicular to the substrate. The dependence of the PL intensity on the rotation angle was recorded and observed to demonstrate the single-crystalline property of the as-prepared wires (Fig. 1f).

### Preferential nucleation and growth along pillar sidewalls

**Figure 2:**
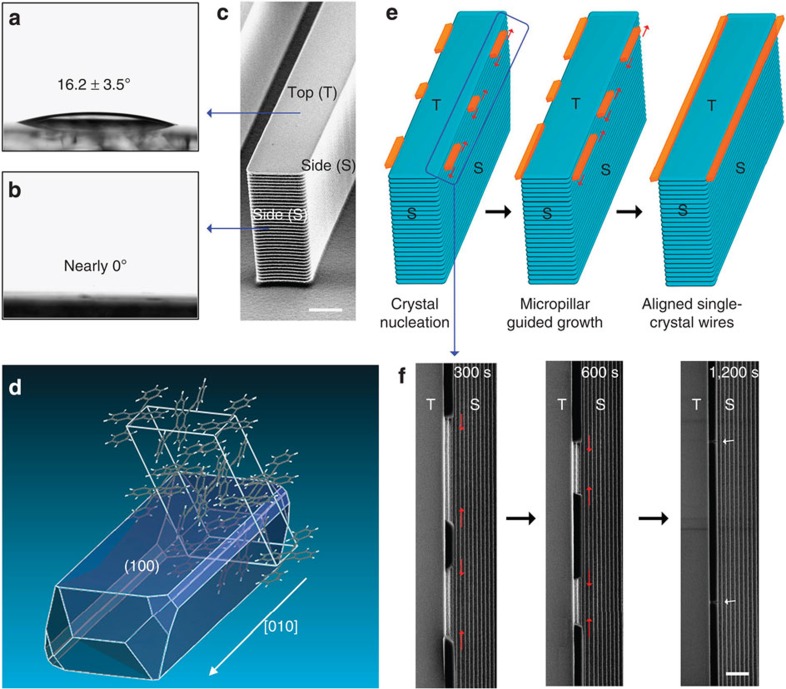
Preferential nucleation and growth. (**a**,**b**) Water contact-angle measurements on the pillar tops and sidewalls. Because of the high roughness combined with the hydrophilic nature of silicon, the pillar sidewall surfaces exhibited CAs as low as 0°, whereas the flat top regions exhibited CAs of 16.2±3.5°. (**c**) An scanning electron microscope (SEM) image of one micropillar, showing the flat top and rough sidewalls fabricated by the stepwise etching process. (**d**) Schematic illustration of the monoclinic, crystallographic structure of a single crystal of BPEA, demonstrating that the preferential growth direction is along the [010] direction. (**e**) Schematic illustration and (**f**) SEM observations of the time-dependent crystal nucleation and growth of BPEA wires anchored to the top edges of the micropillars. Organic semiconductors selectively nucleated on the top edges of the pillars and formed dispersive crystal seeds. Because of the continuous mass influx from the bottom high-temperature zone, the crystals continued to grow horizontally and finally formed aligned organic 1D structures along the pillar edges. Scale bars, **c** 5 μm, **f** 2 μm.

Controlling the position and alignment of single-crystal 1D structures presents several challenges. A major one is the controlling of precise nucleation of the organic semiconductor during the PVT process^1^,^2^,^3^,^4^,^5^,^6^. Generally, organic vapours flow randomly over flat substrates, often leading to the formation of irregular nucleation points, which then grow in every direction and form disordered wires (Supplementary Fig. 5). It is worth noting that microstructured surfaces not only guide liquid flows^25^ but also affect vapour flows^29^. Linear micropillars on silicon substrates have two distinct surfaces, flat tops (T) and rough sidewalls (S), as a result of the physically layer-by-layer etching process (Fig. 2c). Because of the considerable roughness of 584±122 nm and the hydrophilic nature of silicon, the pillar sidewall surfaces in our study exhibited contact angles (CA) as low as 0°, whereas the flat tops exhibited CAs of 16.2±3.5° (Fig. 2a,b). This distinct difference in surface energy between these two surface regions of the micropillars would lead to preferential nucleation of BPEA molecules on the pillar sidewalls. To investigate the wire growth details, scanning tunnelling microscopy (STM) has been used to observe the stacking of first-few-layer BPEA molecules on the silicon surface (Supplementary Fig. 9a). The BPEA molecules show a periodic stacking with m, 5.2±1 Å, n, 16.6±1 Å, θ, 97±1°, which is consistent with the (100) face revealed by single-crystal X-ray diffraction (SCXRD) result of BPEA single crystals (Fig. 2d, Supplementary Table 1 and Supplementary Data 1). These results indicate that the BPEA molecules firstly stack on silicon substrate with (100) plane, then grew along the [010] direction when considering the TEM result (inset image in Fig. 1d).

By virtue of the continuous mass transport from the bottom high-temperature zone to this low-temperature region, these nucleation sites (BPEA islands) grew along the linear micropillars via the kinetically controlled Stranski–Krastanov mode (Supplementary Fig. 11), yielding aligned crystal wires (Fig. 2e). This process was confirmed by detailed, time-dependent observations of the growth behaviour (Fig. 2f). Initially, BPEA molecules selectively nucleated at the top edges of the pillars and formed dispersive seeds. Subsequently, the BPEA seeds continued to grow along the axis direction and formed aligned organic wires along the pillar edges (Supplementary Fig. 2). To investigate the details of the fused region contributed by two independently nucleated domains, we used TEM as well as SAED to study their crystallization information (Supplementary Fig. 10). Different from the well-known Eshelby-twist mechanism elucidated by Jin. *et.al*.^30^,^31^,^32^,^33^, the crystallization in the fused regions of the BPEA wires are similar without a twisted structure.

Diverse experimental parameters have been utilized to control the BPEA wire growth in the GPVT process. The surface energies of the micropillars were tailored to investigate their contribution to the crystal growth (Supplementary Fig. 12). Low surface energy heptadecafluorodecyltrimethoxysilane (FAS) was applied to the completely cover the pillars. After FAS modification, BPEA crystals have grown on both pillar top and sidewall regions. Because the surface energies of the pillar tops were reduced by the FAS modification, the organic semiconductor was deposited with round shapes rather than linear structures (Fig. 1b, c), indicating that the surface energy played a role in the growth of the BPEA wires. Furthermore, vacuum, rather than nitrogen or atmosphere environment, has been used to repeat the similar growth process (Supplementary Fig. 13). However, no BPEA wires formed on the micropillars. The heights of the aligned wires could be tuned by tailoring the initial BPEA concentration, as shown in Supplementary Fig. 14. A dilute BPEA solution resulted in limited mass transport during the GPVT process. A height of 483±22 nm could thus be generated at a concentration as low as 0.25 mg ml^−1^, and the height increased to several micrometres at 2 mg ml^−1^. Furthermore, tailoring the heating rate can affect the flow rate along the micropillars, facilitating the control of the wire height (Supplementary Fig. 15). For a heating rate as low as 0.2 °C per minute, the heat flow was correspondingly weak, yielding a small wire height of 383±21 nm. Notably, this wire height could be increased to ~1.8 μm when the heating rate was increased to 2 °C per minute.

### Controlled joining of wires

**Figure 3:**
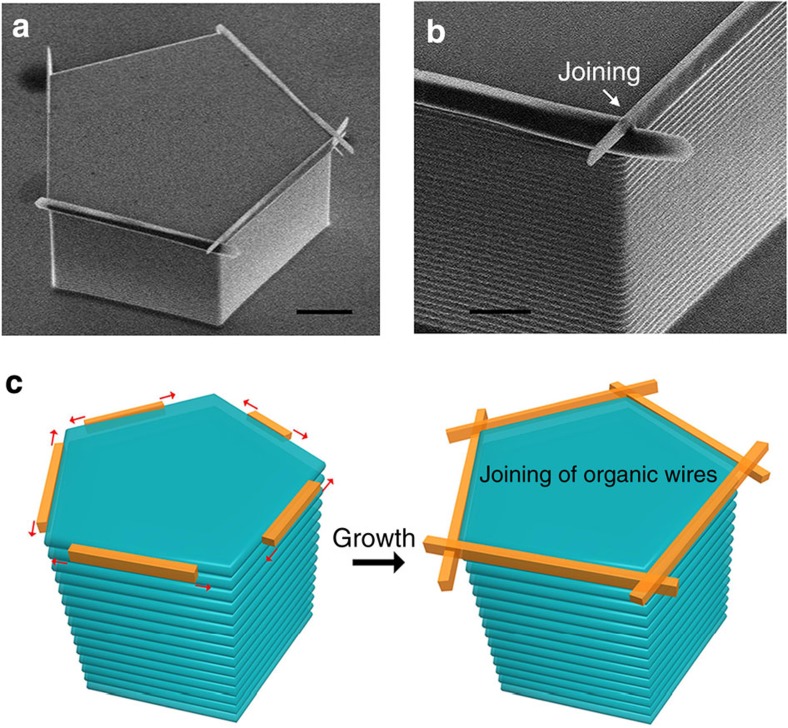
Cross-grown BPEA wires contributed by pentagon-shaped micropillars. (**a**) Scanning electron microscope observation of wire patterns on a pentagon-shaped micropillar. (**b**) Magnified image of **a**, showing that cross-grown BPEA wires dominated by the external angle structure of this micropillar. (**c**) Schematic illustrations of growth of joined wire patterns. BPEA molecules nucleated at each edge of this pentagonal type micropillar, then grew along the pillar edges. Consequently, two growing organic wires met at the corner of the pillar, and joined within a certain internal angle. Scale bars, **a** 10 μm, **b** 2 μm.

Because organic wires exhibit edge-growth behaviour in the GPVT process (Fig. 2), modifying the pillar geometry, such as using polygons instead of lines, allows for the creation of joined organic 1D structures with precise internal angles and positions. Taking a pentagonal type micropillar for an example, joined BPEA wires with 108° internal angles could be fabricated (Fig. 3a). From the magnified image (Fig. 3b), we can find the organic wires were overlapped with each other. The possible growth process was illustrated in Fig. 3c. BPEA molecules nucleated at each edge of this pentagonal type micropillar, then grew along the pillar edges. Consequently, two growing organic wires met at the corner of the pillar, and joined within a certain internal angle.

### Waveguiding ability of single-crystal BPEA wires

**Figure 4:**
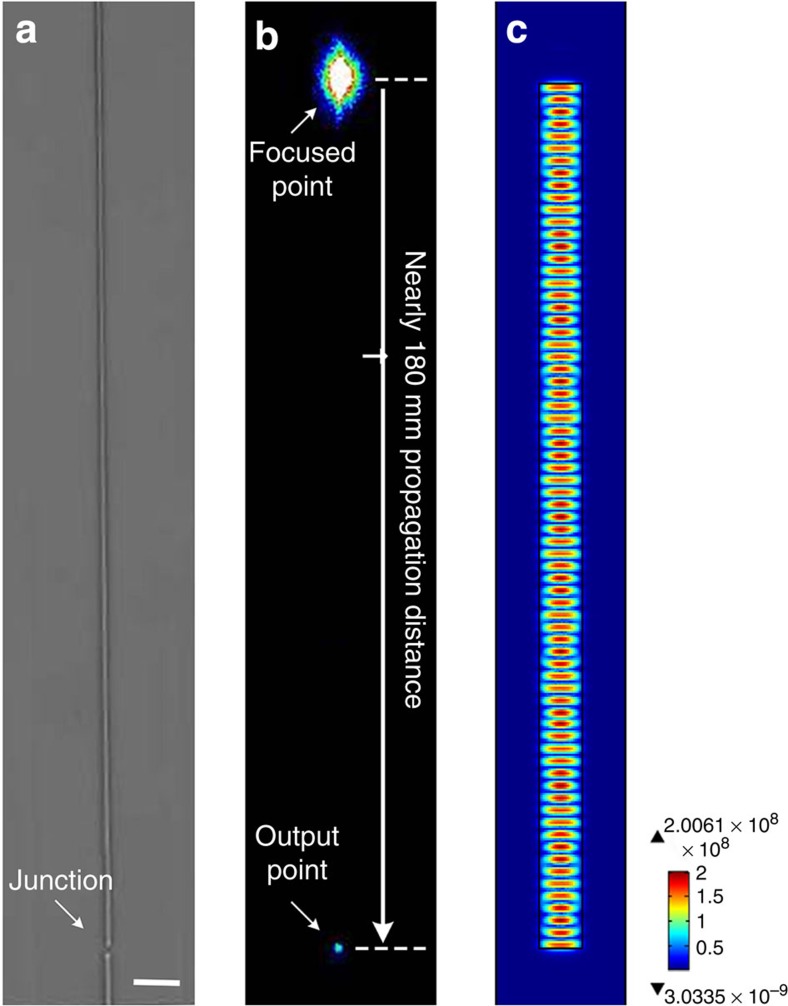
Waveguides along the straight BPEA wires. (**a**) Bright- and (**b**) dark-field optical micrographs of a single-crystal BPEA wire with a length of nearly 200 μm. When a focused laser beam was positioned on the organic linear structure, we clearly observed an output light spot at the junction point, indicating considerable optical waveguiding ability over a propagation distance of nearly 180 μm with a transport loss of 0.0131, dB μm^−1^. (**c**) Simulated electric field intensity distribution (surface: electric field norm (V m^−1^), *λ*=488 nm, *n*=1.80) in the BPEA wire. The regular electric field intensity reveals that the energy of the electric field can be well confined in the architecture as exciton polariton resonators. Scale bar, **a** 10 μm.

Aligned single-crystal organic wires can be utilized in diverse optoelectronic applications^1^,^2^,^3^,^4^,^5^,^6^, herein we show proof-of-principle demonstrations in the waveguiding field. The aligned, single-crystal BPEA wires on a flat PDMS film that were prepared in this study exhibited bright luminescence (Supplementary Fig. 16a,b). A 488-nm laser beam was then focused onto these wires (setup details are provided in Supplementary Fig. 16d) to investigate their waveguiding ability. Notably, visible luminescence spots appeared at each broken point of the BPEA wires, indicating that focused light propagated along these organic 1D structures (Supplementary Fig. 16c). This optical propagation performance can be attributed to the single-crystalline nature of the wires because very little waveguiding behaviour has been observed in polycrystalline/amorphous semiconductors^8^. Careful transferral can yield intact single-crystal BPEA wires with lengths of nearly 200 μm (Fig. 4). Combined bright- and dark-field optical micrographs clearly demonstrated that the light wave could follow the organic 1D structure. The propagation distance of the light along a BPEA wire was nearly 180 μm (the optical loss was 0.0131±0.0004, dB μm^−1^; the calculation details are presented in Supplementary Fig. 16e and Supplementary Note 2), significantly longer than the propagation distance in a comparable nanoparticle assembly^34^ and comparable to that in a flexible inorganic^35^ or organic^36^ microfibre. The optical loss is attributed to the intrinsic low-phase refractive index at the BPEA/air interface. It should be noted that a ‘hybrid plasmonics’ approach by covering a thin metallic layer can be used to eliminate the propagation loss in the organic crystals (Supplementary Fig. 17 and Supplementary Note 3). The average loss can be effectively reduced to 0.00121±0.00009, dB μm^−1^, which corresponds to a loss of <20% for 1 mm of propagation.

### Controllable waveguides of patterned BPEA wires

**Figure 5:**
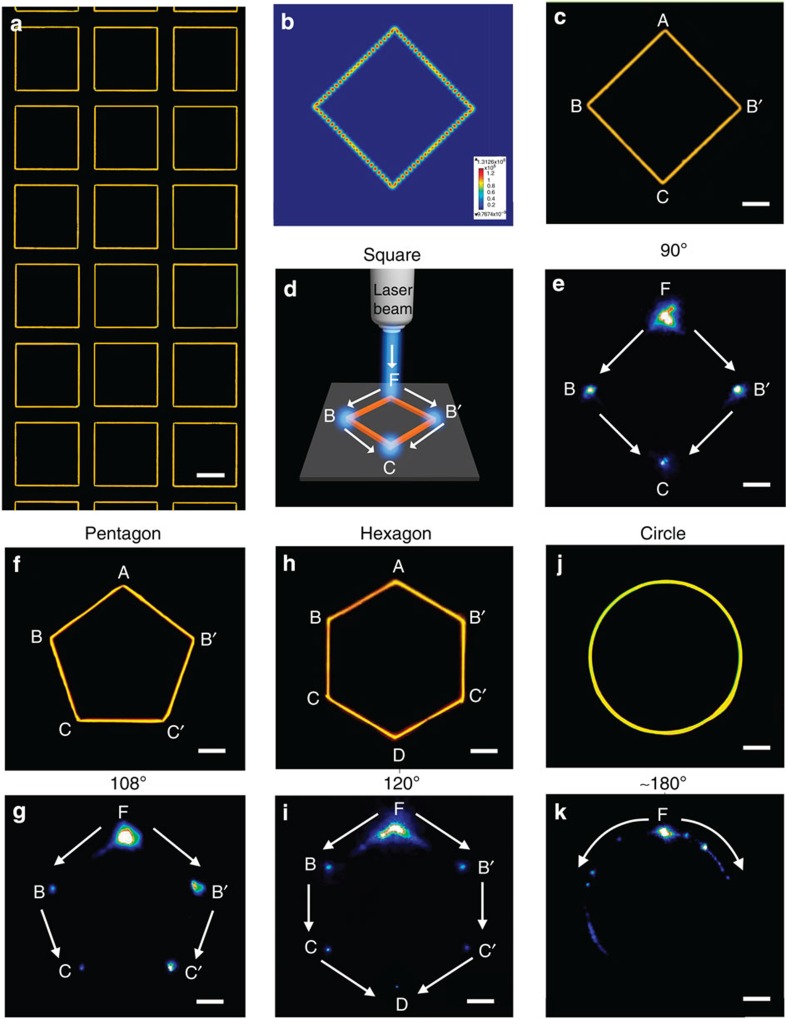
The controlled joining of organic single-crystal wires. (**a**) Dark-field fluorescent micrograph of an array of 90° joined organic wires formed on square-structured micropillars with a pillar length of 40 μm. Dark-field fluorescent micrographs of single (**c**) square, (**f**) pentagonal, (**h**) hexagonal and (**j**) circular BPEA wires, demonstrating that organic, 1D structures can be grown at precise internal angles of 90° (square), 108° (pentagon), 120° (hexagon) and nearly 180° (circle, technically a super-polygon). (**b**) Simulated electric field intensity distribution (surface: electric field norm (V m^−1^), λ=488 nm, *n*=1.80) in the 90° joined BPEA wires. The electric field intensity in each bent section indicates that the energy of the electric field can be well confined in the structure as exciton polariton resonators. (**d**) Schematic illustration of optical waveguiding along square-patterned organic wires. A laser beam was focused on the wires to investigate the optical propagation. (**e**,**g**,**i**,**k**) PL images of waveguiding wire patterns under laser excitation. A 488-nm continuous-wave argon-ion laser was focused to a beam spot size of 1 μm to excite the aligned organic single-crystal wires at an excitation power of <2 W cm^−2^. The light could waveguide through small radii of curvature, for example, internal angles of 90°, 108°, 120° and nearly 180°. A luminescence spot appeared at each joint of the wire patterns, indicating some optical loss in these regions. This effect was particularly strong in the circular wire pattern. Scale bars, **a** 20 μm, **c**,**e** 10 μm, **f**–**k** 10 μm.

Besides pentagonal type micropillars, square, hexagonal and even circular micropillars have been designed to encourage the joining of organic single-crystal wires. Using a well-defined micropillar design, square BPEA wire patterns, for example, can be generated on a large scale, as shown in Fig. 5a. A representative dark-field fluorescent micrograph depicts BPEA wires joined at internal angles of 90° (Fig. 5c), and their waveguiding potential was simulated using the 3D finite element method. The electric field intensity in this wire pattern on a flat substrate after excitation at 488 nm is presented in Fig. 5b. The electric field intensity at the 90° joined sections indicates that the energy of the electric field can be well confined in this structure as exciton polariton resonators and that scattering into the air and the substrate is quite limited. Then, the waveguiding behaviour of these joined organic wires was studied using far-field microscopy and spectroscopy. Representative data collected from this on-demand designed, square BPEA wire pattern are presented in Fig. 5e. When we focused a continuous wave laser onto one joined region of this BPEA wire pattern (Fig. 5d), the generated PL was strongly guided by the regularly arranged structure, causing the light to waveguide through a small radii of curvature (~90°). In addition to square wire patterns, pentagons (Fig. 5f,g and Supplementary Fig. 18), hexagons (Fig. 5h,i), and even circles (technically super-polygons, Fig. 5j,k) of joined BPEA wires could all be generated, which enable light to waveguide within the diverse small radii of curvature, such as 108°, 120° or even nearly 180°.

### Propagation loss caused by cross-joined BPEA wires

It should be noted that a luminescence spot appeared at each corner in these square, pentagon and hexagon patterns, indicating some optical loss at these points^37^. Because of the overlap of the BPEA wires in the joining regions on the pentagon-shaped micropillars (Fig. 3), some of the light was dissipated into the atmosphere instead of being transported inside the organic wires. As a result, the total optical loss increased to 0.0531±0.0012, dB μm^−1^ because of these crossed wire structures (the detailed calculations of the optical loss in the corner regions are presented in Supplementary Note 4). This value is >4 times greater than the value of a straight section that is free of wire joining 0.0131±0.0004, dB μm^−1^). This trend is most obvious in the circular BPEA wire pattern. A circle can be regarded as a super polygon with numerous convex corners. Therefore, the as-prepared BPEA wires in the circular pattern possessed considerable surface defects (that is, overlap or insufficient growth in the joining parts, as shown in Supplementary Fig. 19), which caused the guided light to scatter into a series of bright spots along the pattern (Fig. 5k). Consequently, the propagation distance along the circle-shaped wire pattern was greatly reduced, yielding considerable optical losses of 0.0924, dB μm^−1^.

### Interior angle-mediated joining of wires

**Figure 6:**
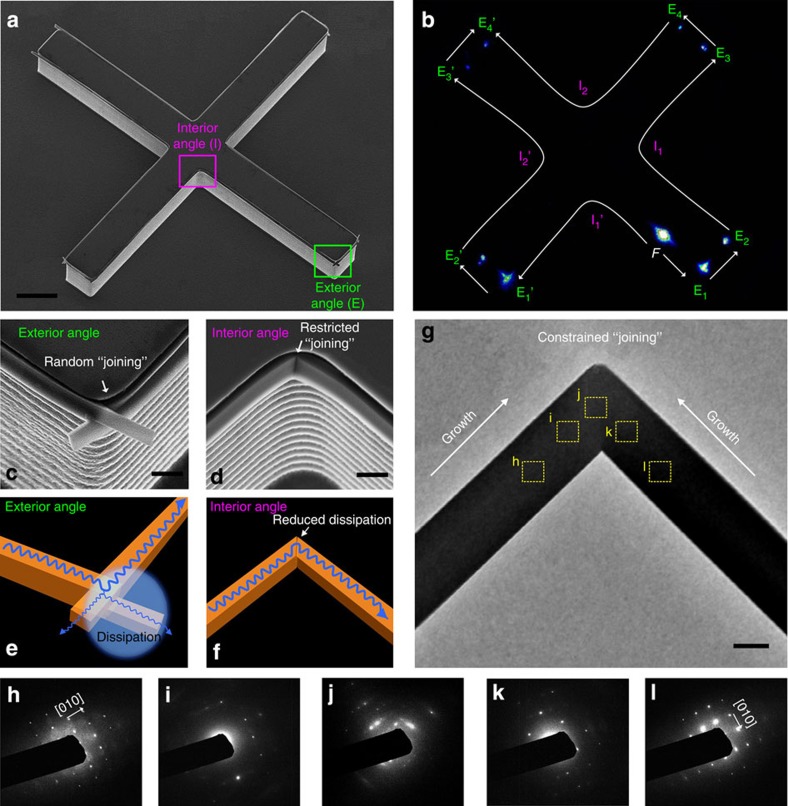
Interior angle-mediated joining of wires to reduce the optical transport loss. An ‘X’-shaped micropillar, consisting of both 90° interior and exterior angles, was used to control the joining of BPEA wires. (**a**) Scanning electron microscope (SEM) observation of the BPEA wire joining on the ‘X’-shaped micropillar. (**b**) PL image of the waveguiding in a wire pattern grown using the ‘X’-shaped micropillar under laser excitation. The lights can waveguide through small radii of curvature of ~90° joints of the wires. It should be noted that the regions with interior angle-mediated wire joining (pink ‘*I*’ points) are dark (no visible luminescence), whereas obvious spots are apparent at each exterior angle-mediated wire joint (green ‘*E*’ points). This result indicates that interior angle-mediated wire joining offers improved optical waveguiding at precise internal angles with low loss. SEM images of (**c**) exterior and (**d**) interior angle regions marked in **a**. Schematic illustration s of the optical waveguiding along the (**e**) cross-grown and (**f**) merged wire joints, which are corresponding to exterior and interior angle joint wires shown in **c** and **d**, respectively. (**g**) TEM image of 90° joined wires. (**h**–**l**) SAED patterns captured from the area marked in **g**. These SEAD patterns show an ordered–disordered–ordered evolution of crystallization around the ‘joining’ area, which demonstrated that the ‘joining’ area has a buffer zone with gradually changed molecular orientation. Scale bars, **a** 5 μm, **c**,**d** 1 μm, **g** 500 nm.

Exterior angle-shaped micropillars (Fig. 5) enable the joining of BPEA wires with controlled angles, allowing for on-demand waveguides through a small radii of curvature at the cost of considerable optical loss. As an alternative method, interior angle-shaped micropillars would allow for the improvement of the joining of the BPEA wires. In this case, the light could waveguide through the small radii of curvature not only at the designed angles but also with reduced optical loss. Therefore, an ‘X’ shaped micropillar, consisting of both 90° interior angles and exterior ones, was utilized to guide the joining of BPEA wires (Fig. 6a and Supplementary Fig. 20). When we focused a laser beam on this pattern (Fig. 6b), the generated PL was strongly guided by the regularly arranged structures, allowing the light to waveguide through the small radii of curvature at angles of 90° along both the interior and exterior angles. Notably, the regions around the interior angle-mediated wire joints (pink ‘*I*’ points) were dark without any visible luminescence, whereas obvious spots were visible at each exterior angle-mediated wire joint (green ‘*E*’ points). The optical loss in the interior angle regions was calculated to be 0.0243±0.0005, dB μm^−1^, and that in the exterior angle regions was 0.0767±0.0012, dB μm^−1^ (the detailed calculations of the optical loss in corner regions are presented in Supplementary Fig. 21c,f). This result indicates that interior angle-mediated wire joining offers improved optical waveguiding at precise internal angles with a low loss.

By comparing the magnified observations of an interior angle region (Fig. 6d) and its exterior angle counterpart (Fig. 6c), we identified two different joining models for the BPEA single-crystalline wires. The BPEA molecules nucleated at each sidewall of the micropillar, grew and then met at the corner. Cross-stacked wires were observed (Supplementary Fig. 21d) at exterior the angle region, similar to the joining behaviour observed on the pentagon-shaped micropillar (Fig. 4). However, due to the geometrical confinement effect, the organic 1D structures combined into an integrated region at interior angle region, as seen in the TEM image (Fig. 6g). When they merged in the 90° interior angle region, a smooth joining without obvious grain boundary of these two wires was observed. To fundamentally understand the crystallization of joint wires, crystalline evolution around the ‘joining’ area was investigated through a series of SAED characterizations (Fig. 6h–l) on the position marked in Fig. 6g. Firstly, clearly observed SAED spots and their square symmetry in two domains of h and l (Fig. 6h,l) suggest that the main-bodies of wires are single-crystalline phase. Then, diffraction spots become relatively disordered and could not correspond to one set of the BPEA single-crystalline lattice (Fig. 6i,k), when focused on the domains closer to the centre of the joint (domains of i and k marked in Fig. 6f). Finally, SEAD spots are totally disordered at the centre of ‘joining’ area (Fig. 6j). The disordered diffraction spots at the junction area demonstrate that large amount of defects are emerged to buffer the changed crystallographic orientation. These results show an ordered–disordered–ordered evolution of molecular stacking around the ‘joining’ area. In detail, the molecular stacking is highly ordered in the main-body of BPEA wires (Fig. 6h), then slightly disordered stacking (Fig. 6i) emergence when is closer to the centre, followed by totally disordered stacking (Fig. 6j) at the centre, which can also gradually change to ordered stacking state with 90° rotation of crystallographic orientation (Fig. 6k,l) in the process of moving away from centre. Therefore, joining of wires attributed to a buffer zone with a gradually changed molecular-stacking orientation. Meanwhile, the bright spots of Fig. 6h–j indicate the good crystallinity at this restricted ‘joining’ position, which contributes to the effective flow of photons with low loss at this interior corner. In this investigation, we create an effective technique for controlling the joining of organic wires, yielding reduced optical loss.

### Wide adaption for different molecules

The GPVT technique provides unique advantages for the single-step crystallization and alignment of various organic single crystals because preferential nucleation and growth are the commonly encountered problems in the alignment of various structures. We also tested our ability to achieve controlled growth of other molecules (Supplementary Fig. 22), including di-2,5-bis(dodecylthiophene)-thieno[3,2-b]thiophene, 1,4-dimethoxy-2,5-di[4’-(methylthio)styryl]benzene.

## Discussion

The tunable joining of organic single-crystal wires is of great importance in optoelectronic application; however, few attempts^9^,^10^,^11^,^12^,^13^,^14^,^15^,^16^,^17^,^18^,^19^,^20^ have been made to achieve this feat because of the difficulty of controlling the crystal nucleation and growth. Only a few studies to date have reported dewetting-induced curved^35^,^36^, adhered^37^ or dendritic organic 1D nanostructures^38^, however, controlling the joining angles of wires is still an open question. Our strategy provides an effective method of controlling the nucleation and growth of organic crystals to precisely dictate the joining of organic 1D structures.

We have demonstrate a facile and effective GPVT technique to control the growth, alignment and positioning of organic single-crystal wires. Hundreds of micrometres-long, precisely positioned, highly aligned and aligned organic single-crystal wire arrays can be generated. Besides regular linear arrangements, organic single-crystal wires can be joined with precisely controlled angles by tuning the pillar geometries, which allows light to waveguide through small radii of curvature. A high-crystalline buffer zone with gradually changed crystallographic orientation was discovered at the restricted ‘joining’ area, which provides new understanding on organic wire joining mechanism and methodology for performance optimization of waveguides. Our technique offers the possibility to construct more complex organic linear structures and will lead to advancement in the growth-by-design approach in the fabrication of organic single-crystal semiconductors towards low-cost and high-performance optoelectronic applications.

## Methods

### Fabrication of pillar-structured substrates

Silicon wafers (10 cm in diameter, N doped, <100> oriented, 525 μm in thickness) were structured using a direct laser-writing apparatus (Heidelberg DWL200) that transferred the computer-predefined design onto the photoresist (Shipley Microposit S1800 series)-coated wafer with ~1-μm precision. After irradiation and development, the wafers were etched using deep reactive-ion etching (DRIE, Alcatel 601E) with fluorine-based reagents for various times (10 s–6 min) depending on the desired height of the structures. Pillar-structured silicon substrates with tunable pillar top areas, pillar gaps and pillar top shapes could be fabricated. After resist stripping (Microposit Remover 1165), the substrates were cleaned using ethanol and acetone. To tailor the wetting properties of the micropillars, the as-prepared silicon substrates were silanized using FAS in a decompression environment at room temperature for 24 h and then heated at 80 °C for 3 h, resulting in reproducible, homogeneous, low surface energy surfaces.

### GPVT technique

Pillar-structured substrates, instead of flat surfaces such as those used in a traditionalPVT system, were employed to encourage preferential nucleation and growth of the organic semiconductor and to yield aligned single-crystal wires. In a typical procedure, BPEA was dissolved in ethanol (0.25–2 mg ml^−1^) and placed in a manually formed square boat (1 × 1 × 1 cm^3^) consisting of tinfoil with an open top. This metal boat, loaded with ~100 μl of BPEA solution, was placed on a heating stage at 453 K (above the evaporation temperature of ethanol and near the sublimation temperature of BPEA). With the rapid evaporation of the ethanol, the BPEA formed a thin layer at the bottom of the metal boat. In contrast to the traditional PVT process, in which aggregated semiconductor powders are directly evaporated, an organic solution was employed in this study to provide a homogenous evaporation resource. A pillar-structured substrate with a rough surface, such as lines, squares, pentagons, hexagons, circles or ‘X’ shapes, was then placed facedown onto the metal boat. A suitable deposition time was found to be 15–30 min. During this process, the BPEA would preferentially nucleate and grow at the pillar edges, yielding precisely positioned organic single-crystal wires. The height of the single-crystal wires could be tuned by modifying the initial BPEA concentration. A dilute BPEA solution resulted in limited mass transport during the GPVT process. The height of the wires could be varied from ~490 nm to over 2 μm. The entire process was performed in a nitrogen-protected sealed box, and could also be processed at atmosphere. The as-prepared organic wires were transferred onto flexible PDMS films through a contact-printing technique to allow for further investigation of the optical waveguiding behaviour. To demonstrate the broad adaptability of this GPVT strategy, other organic molecules, such as di-2,5-bis(dodecylthiophene)-thieno[3,2-b]thiophene and 1,4-dimethoxy-2,5-di(4’-(methylthio)styryl)benzene, were subjected to the same process except for the preparation of the evaporation source.

### Optical waveguides

The optical measurements were performed on a custom-built inverted Olympus FV-1000 microscope by exciting the samples in the ultraviolet band (see details in Supplementary Fig. 16e). A 488-nm continuous-wave argon-ion laser (Spectra-Physics) was focused to a beam spot size of 1 μm to excite the aligned organic single-crystal wire at an excitation power of <2 W cm^−2^. PL images were acquired using a Nikon inverted microscope with a thermal-electrically cooled CCD (Princeton Instruments, ProEm:512B), and micro-area spectra were measured using a Princeton Instrument spectrometer. All experiments were performed at room temperature and at the atmospheric pressure.

### Characterization

The structures of the organic single-crystal wires were investigated via scanning electron microscopy (SEM, Hitachi, S-4800, Japan) at an accelerating voltage of 5.0 kV. The crystallinity of the wires was confirmed using a JEOL TEM-2100 operating at a 200-keV accelerating voltage. To recover the 90° joined wire regions, the ‘X’-shaped micropillars surrounded by the organic wires were broken into pieces. Subsequently, the fragments were dispersed in water and then drop cast onto a copper grid. After a number of TEM observations, we were able to find a few joined wire sections that remained intact. Optical and fluorescent micrographs of the BPEA wires were obtained using an optical microscope (Vision Engineering Co., UK) that was coupled to a CCD (charge-coupled device) camera and connected to a desktop computer. Fluorescence spectra were recorded using an F-4500 fluorescence spectrophotometer (Hitachi, Japan). Static CAs were measured using a Dataphysics OCA20 contact-angle system at ambient temperature. The average CA was obtained by measuring at more than five different positions on the same sample. For the contact-angle measurements, a flat silicon substrate was employed as a representative of the top surface of a micropillar. In total, 20 silicon wafers (500 μm in thickness) were closely stacked and etched on one side from top to bottom. The etched side of each wafer was calibrated to form a rough surface that could be treated as representative of the sidewall of a micropillar. STM experiments were performed on a Nanoscope E STM instrument (Bruker) with a tungsten tip, which was electrochemically etched in 0.6 M KOH. The SCXRD was performed on a Rigaku Saturn724 CCD diffractometer using graphite-monochromatized Mo Kα radiation (*λ*=0.71073 Å) at 173 K. The molecular stacking model is calculated based on the SCXRD results using Materials Studio package^39^.

## Author contributions

Y.W., B.S. and L.J. designed the experiments. Y.W., J.F., X.J., Z.Z. and X.W. performed the experiments and analysis. Y.W., J.F. and B. S. wrote the paper.

## Additional Information

**Accession codes:** The X-ray crystallographic coordinates for structures reported in this Article have been deposited at the Cambridge Crystallographic Data Centre (CCDC), under the deposition number CCDC 1050623. These data can be obtained free of charge from The Cambridge Crystallographic Data Centre via www.ccdc.cam.ac.uk/data_request/cif.

**How to cite this article**: Wu, Y. *et al*. Positioning and joining of organic single-crystalline wires. *Nat. Commun.* 6:6737 doi: 10.1038/ncomms7737 (2015).

## Supplementary Material

Supplementary Information Supplementary Figures 1-22, Supplementary Table 1, Supplementary Notes 1-4 and Supplementary References

Supplementary Data 1 Crystallographic Information File for BPEA.
