# Supplementary material for: Positioning and joining of organic single-crystalline wires
Source: Nat Commun. 2015 Mar 27;6:6737. doi: 10.1038/ncomms7737 (PMC4389254; doi:10.1038/ncomms7737)
Supplement: Supplementary Information — Supplementary Figures 1-22, Supplementary Table 1, Supplementary Notes 1-4 and Supplementary References [file ncomms7737-s1.pdf]

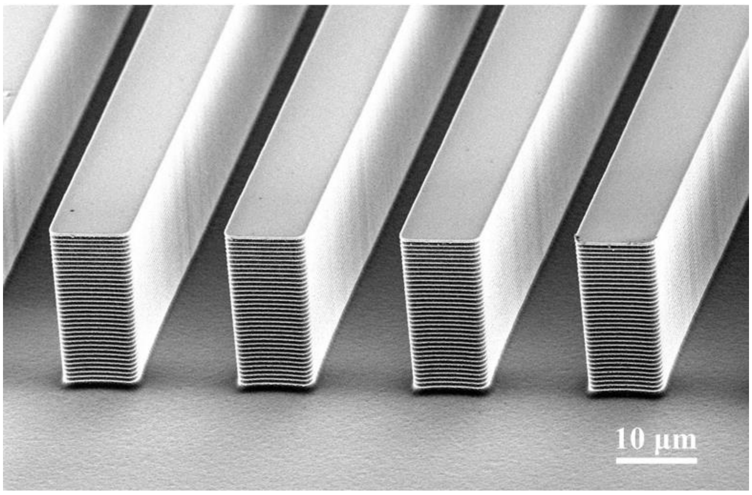

Supplementary Figure 1 | Scanning electron micrograph (SEM) of a groove-structured silicon substrate. The micropillars are *ca.* 10 µm wide, 20 µm high and own the gap of 10 µm.

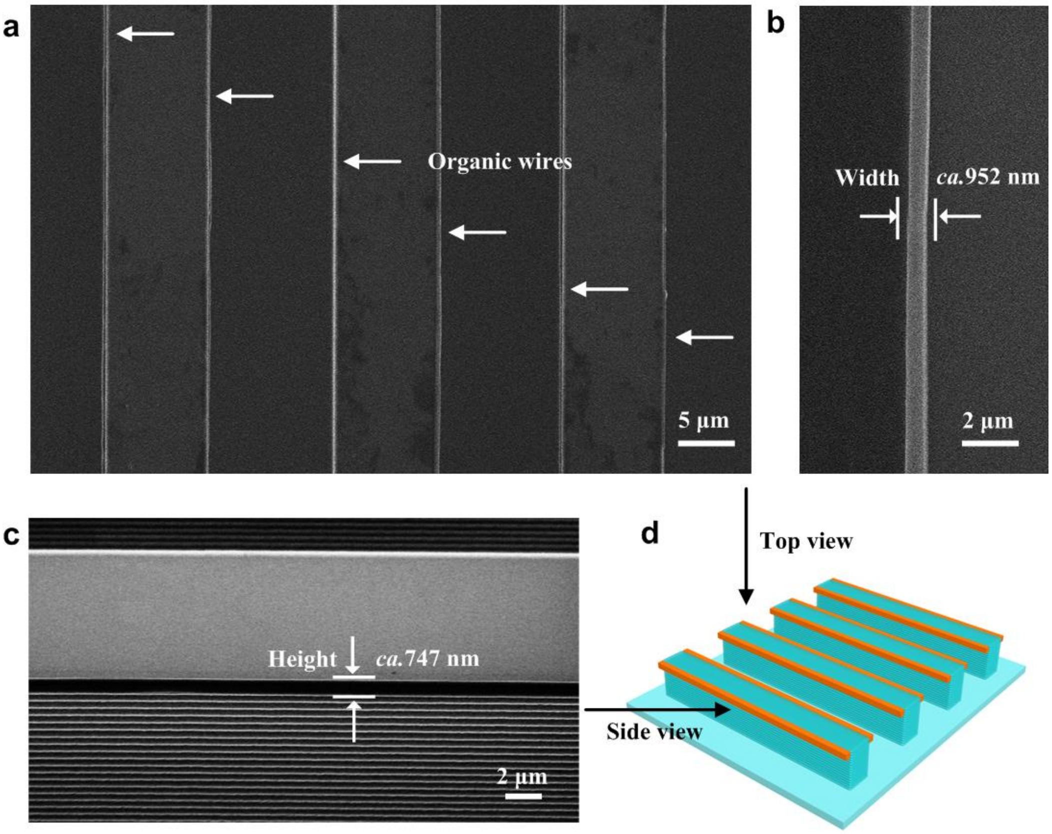

Supplementary Figure 2 | Strictly aligned organic 1D architectures are anchoring to the tops of pillar sidewalls in the guided physical vapor transport (GPVT) strategy. (**a**) Top view and (**c**) 45° side view SEM observation of aligned 9,10′-bis(phenylethynyl) anthracene (BPEA) wires grown upon a groove-structured silicon substrate. Each organic 1D architecture is uniform, continuous, precisely positioning and highly oriented. (**b**) Magnified image of **a** showing the typical width of one BPEA wire. (**d**) Schematic illustration of aligned BPEA wires to show the view angles of the SEM investigations.

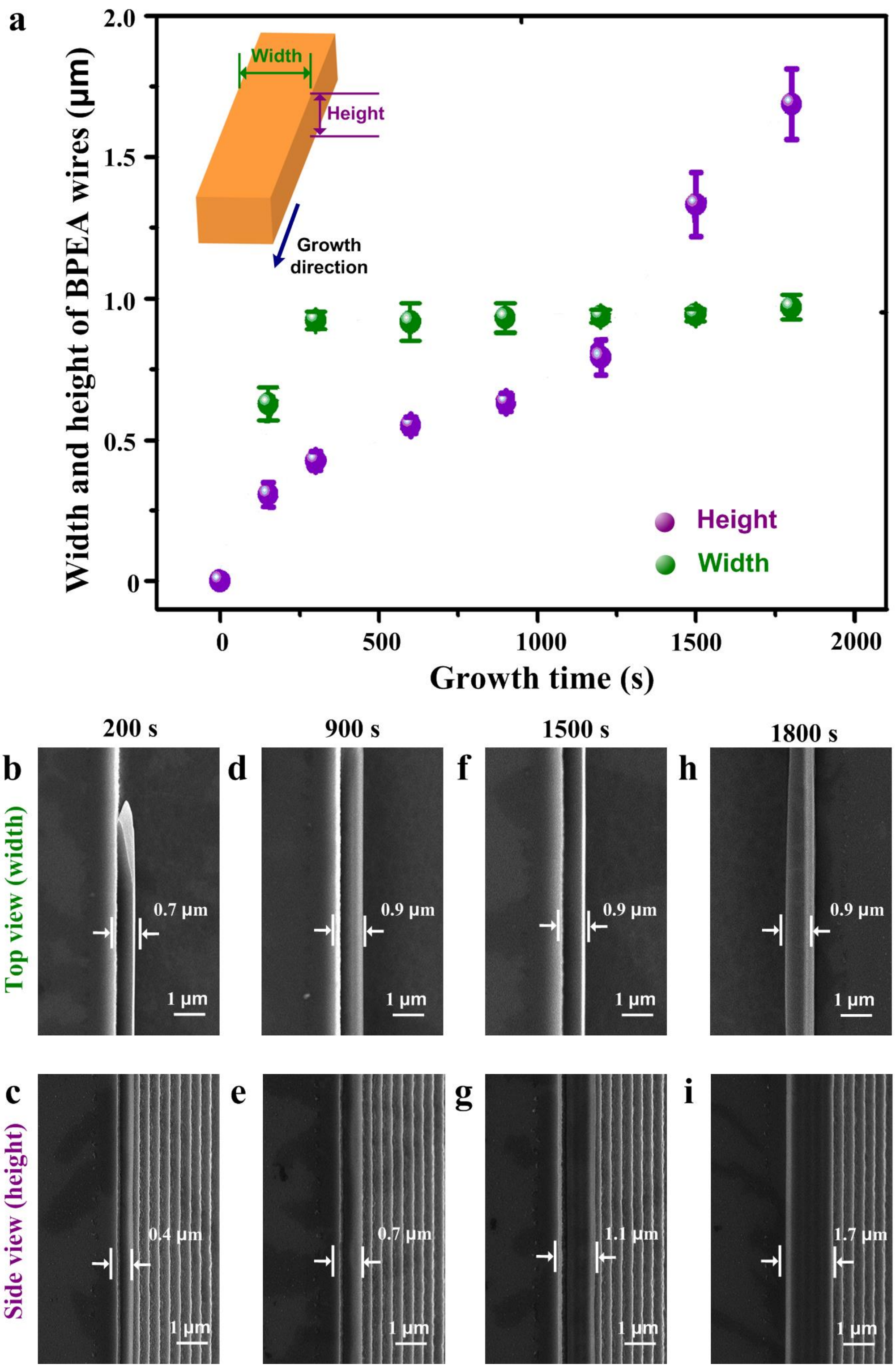

Supplementary Figure 3 | The width of BPEA wires remained while their height increased continuously during the GPVT process. (**a**) The dependence of wire width and height on the growth time. The data at each growth time have been obtained from 50 samples. During the growth of organic 1D architectures, the width of BPEA wires increased then kept stationary. In contrast, the wire height continued to grow. The inset image is schematic illustration of crystallographic structure of a BPEA wire. (**b**, **d**, **f**, **h**) representative top view SEM observations of aligned BPEA wires with diverse widths depending on growth time. (**c**, **e**, **g**, **i**) representative side view SEM observations of aligned BPEA wires with different heights depending on growth time.

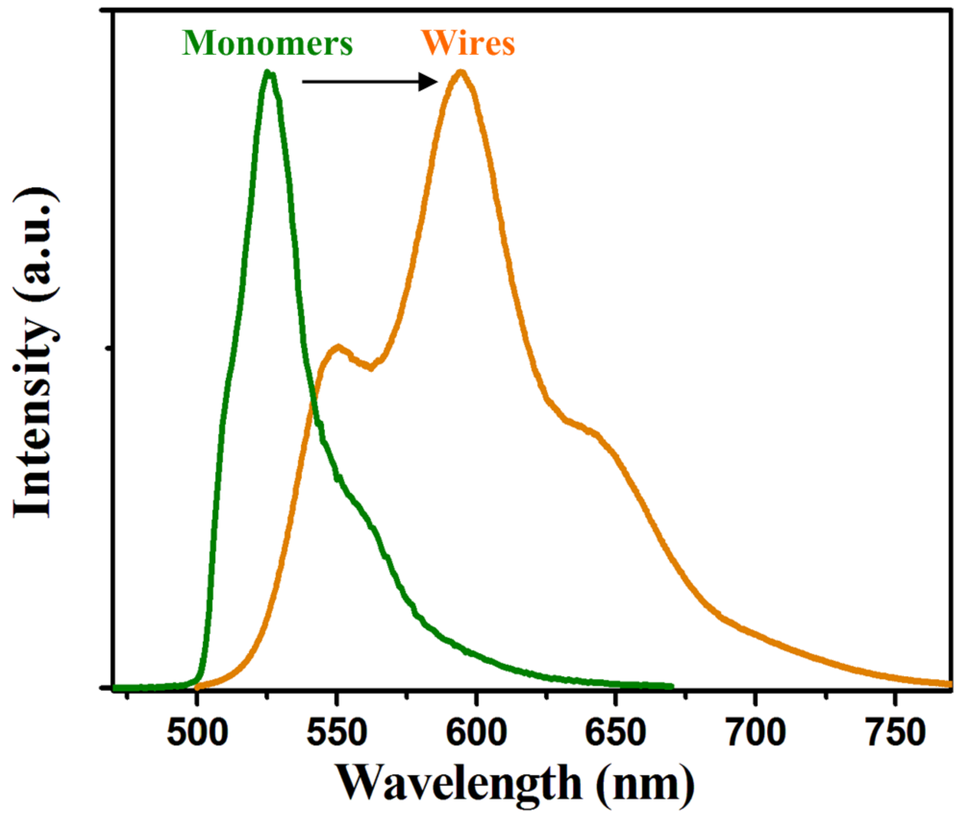

Supplementary Figure 4 | A considerable red-shift of photoluminescence (PL) spectra appeared in aligned 9,10′-bis(phenylethynyl)anthracene (BPEA) wires due to close-packed aggregation of BPEA molecules in the crystal. PL spectra of BPEA monomers in ethanol (left) and aligned BPEA wires (right). The emission peak of BPEA wires shifts by as much as 80 nm with respect to that of the monomers, and the emission color changes from green to yellow.

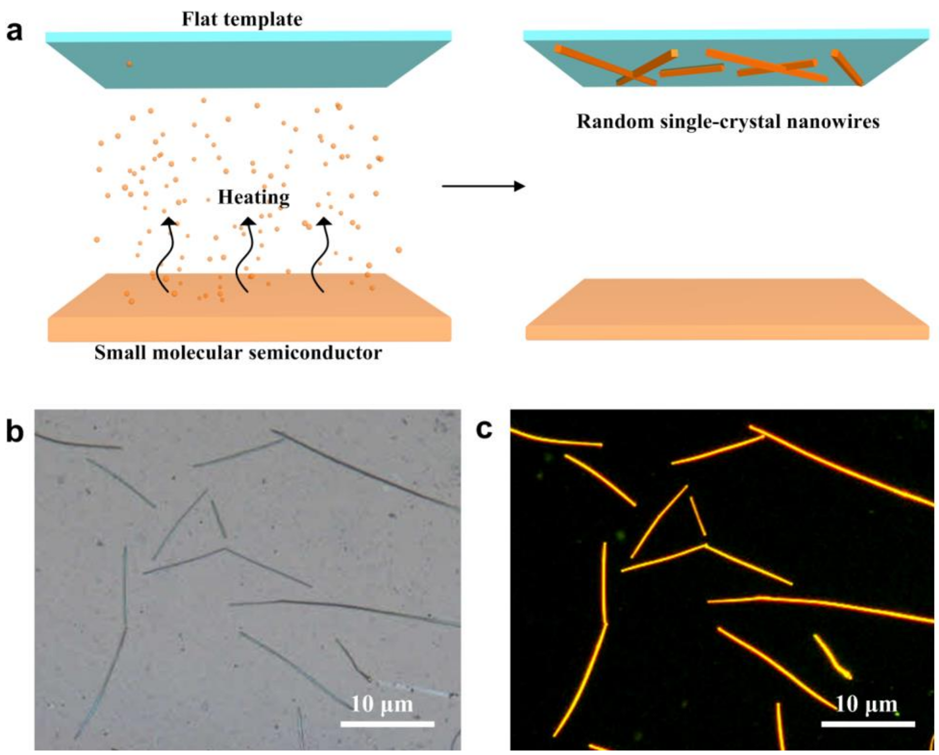

Supplementary Figure 5 | Disordered BPEA wires grown upon a flat silicon substrate due to randomly positioning of nucleation and uncontrolled crystal growth. (**a**) Schematic illustration of the growth of organic 1D architectures upon a flat substrate in the physical vapor transport (PVT) system. The flat surface is unable to encourage the nucleation and growth of organic semiconductor, yielding randomly positioning BPEA wires. (**b**) Optical and (**c**) in-site fluorescent micrographs of disordered BPEA wires grown upon a flat silicon substrate.

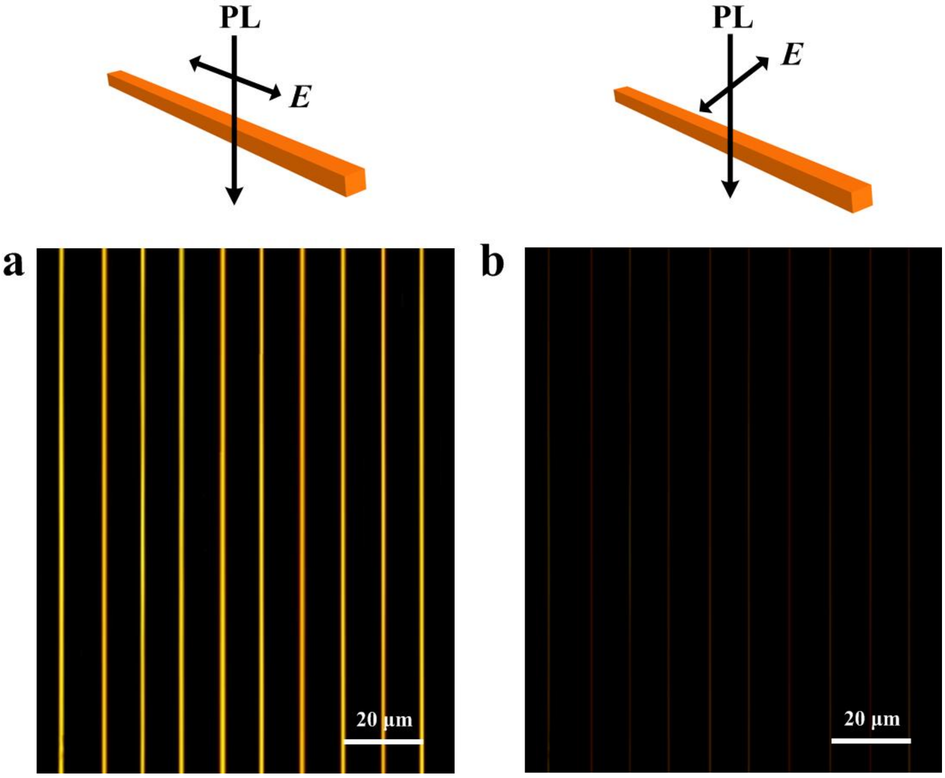

Supplementary Figure 6 | Angle-dependent polarized investigation confirms the single crystalline of as-prepared BPEA wires in the GPVT strategy. Cross-polarized optical micrograph of aligned BPEA wire arrays with (**a**) parallel (180°and 360°) and (**b**) vertical (90° and 270°) to the electric field direction, respectively. The entire wires change from bright to dark simultaneously, on rotating these linear structures about an axis perpendicular to the substrate. The top images are schematic illustrations to indicate the orientation of crossed polarizers.

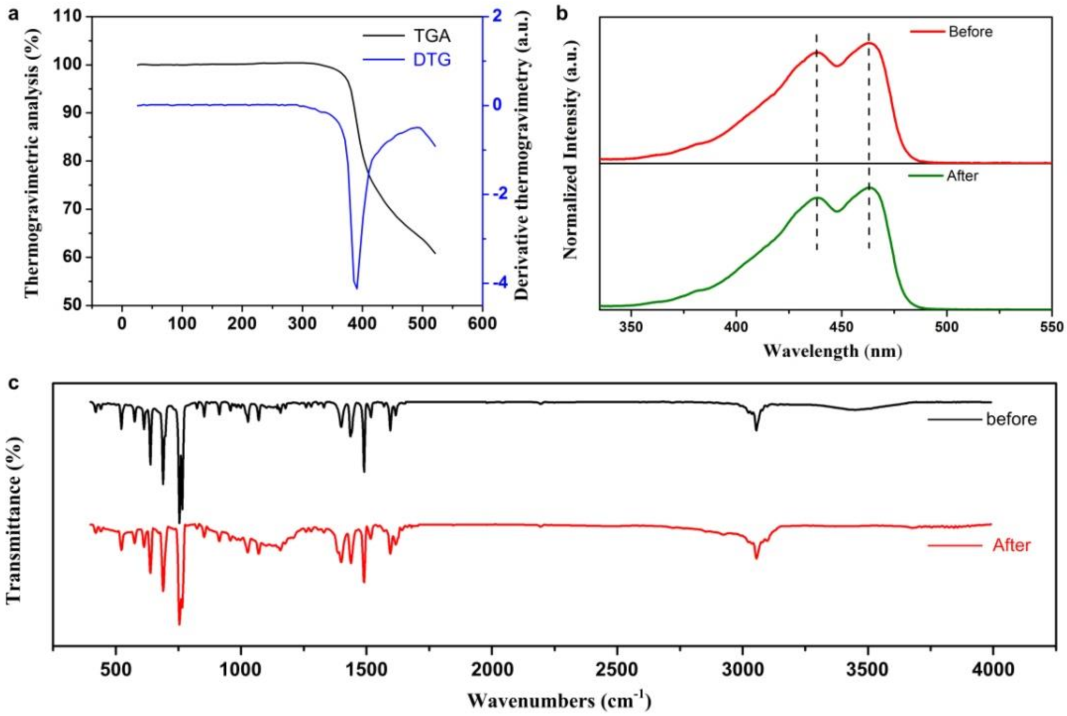

Supplementary Figure 7 | Stable molecular structure of BPEA before/after the GPVT in air. (**a**) Thermogravimetric analysis (TGA) of BPEA. (**b**) Ultraviolet (UV) absorption spectrum and (**c**) infrared spectroscopy (IR) of BPEA before and after heating at 200 °C through the GPVT process in air.

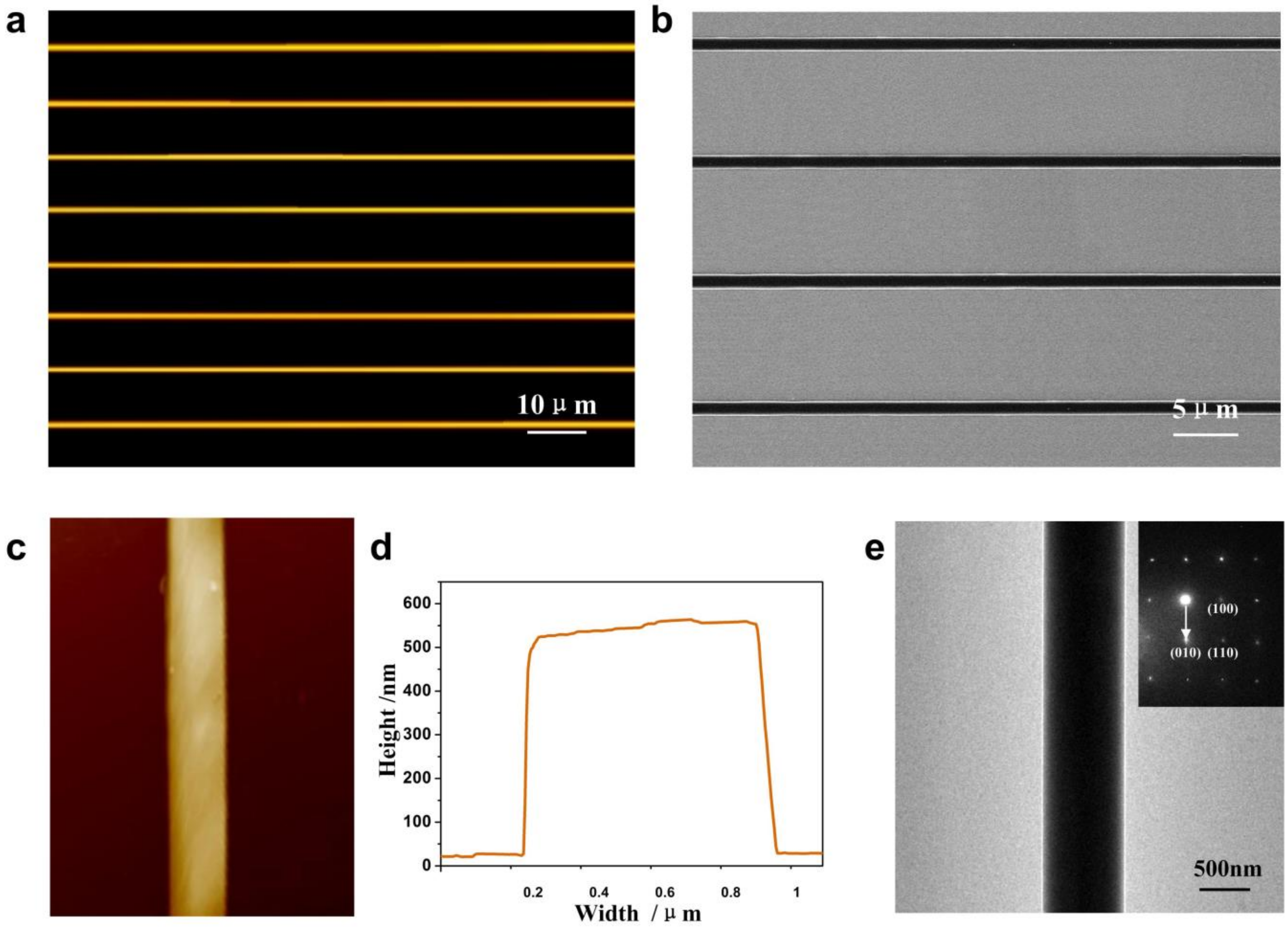

Supplementary Figure 8 | Stable morphology and crystal-structure of BPEA wires grown in air. (**a**) Dark-field fluorescent micrograph exposed at 325 nm UV irradiation and (**b**) SEM image of highly aligned BPEA wires upon a flat polydimethylsiloxane (PDMS) film. Those ordered 1D architectures grown in air also has high uniformity, precise position and homogenous width similar to that grown in nitrogen-protected atmosphere (Fig.1). (**c, d**) AFM characterization of single BPEA wire. The smooth top surface indicates high crystalline degree of as-prepared wires. (**e**) TEM image of BPEA wire grown in air. The inset image is a selected-area electron diffraction (SAED) pattern, which indicates the wire has a single-crystal structure and grow along the [010] direction.

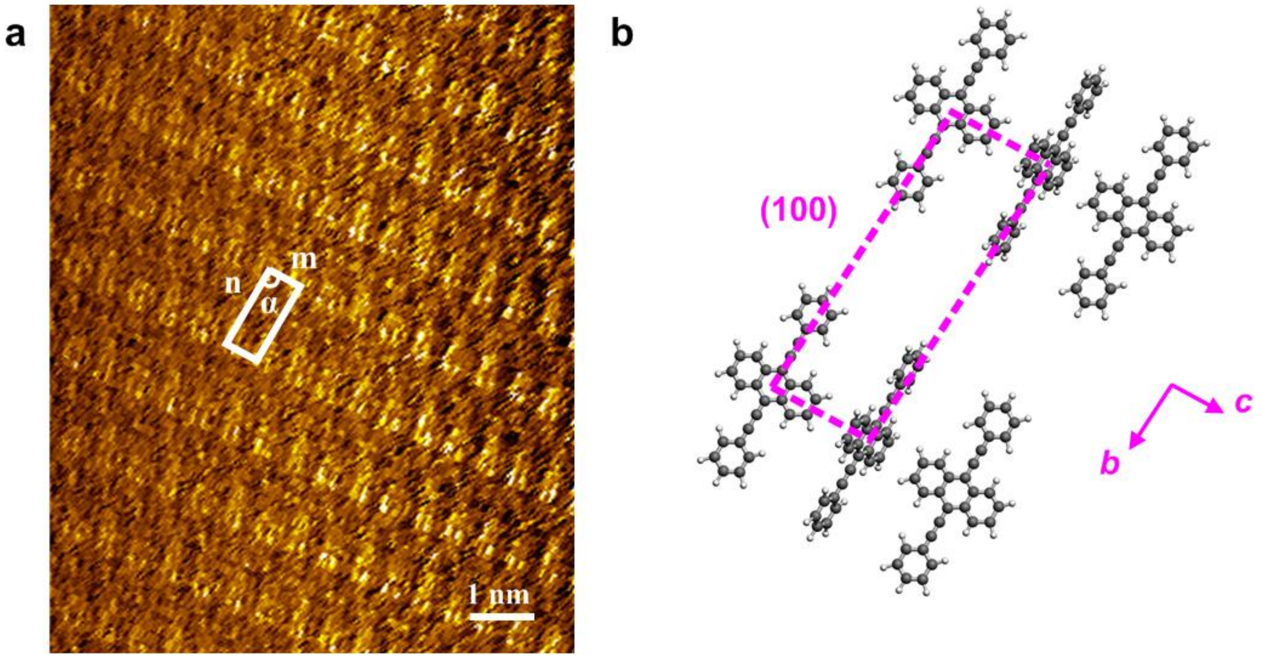

Supplementary Figure 9 | Molecular stacking of first-few-layer BPEA molecule on silicon surface. (**a**) Scanning tunneling microscopy (STM) observation of first-few-layer BPEA molecule on silicon surface. The BPEA molecules show a periodic stacking with m, 5.2 ± 1 Å, n, 16.6 ± 1 Å, θ, 97 ± 1°, which consistent with the (100) face of BPEA single-crystals. (**b**) Schematic illustration of BPEA molecule stacking according to the STM result.

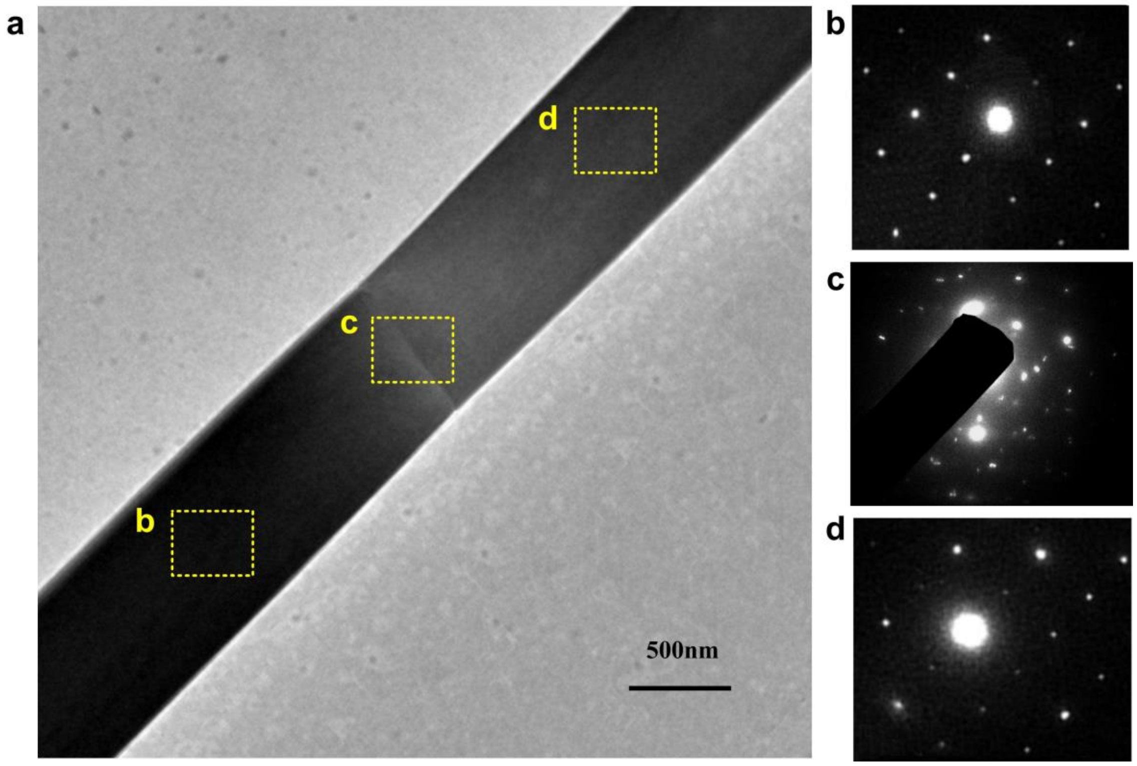

Supplementary Figure 10 | The crystallization in the fused regions of the BPEA wires are uniform without twisted structure. (**a**) TEM image of a BPEA wire with grain boundary. (**b**-**d**) SAED patterns captured from the areas marked in **a**.

a b

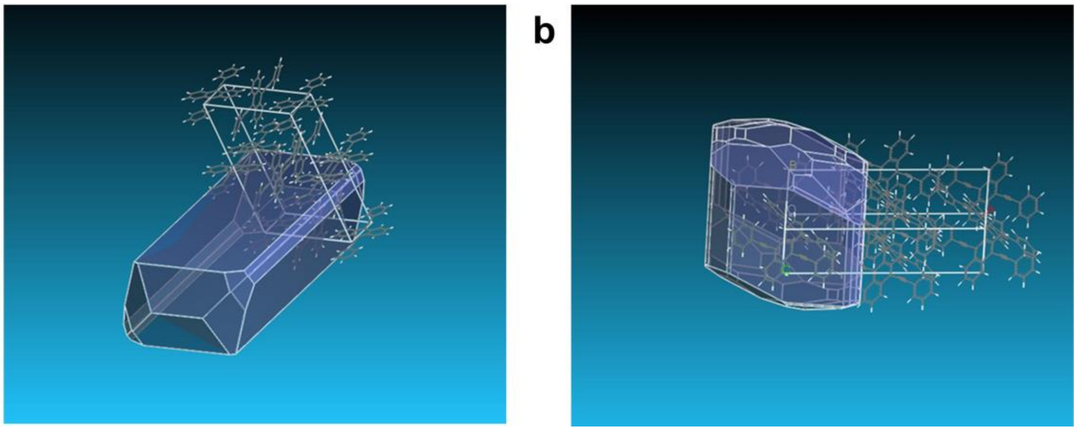

Supplementary Figure 11 | Growth morphologies of (**a**) kinetically-controlled, (**b**) thermodynamically-controlled BPEA crystals.

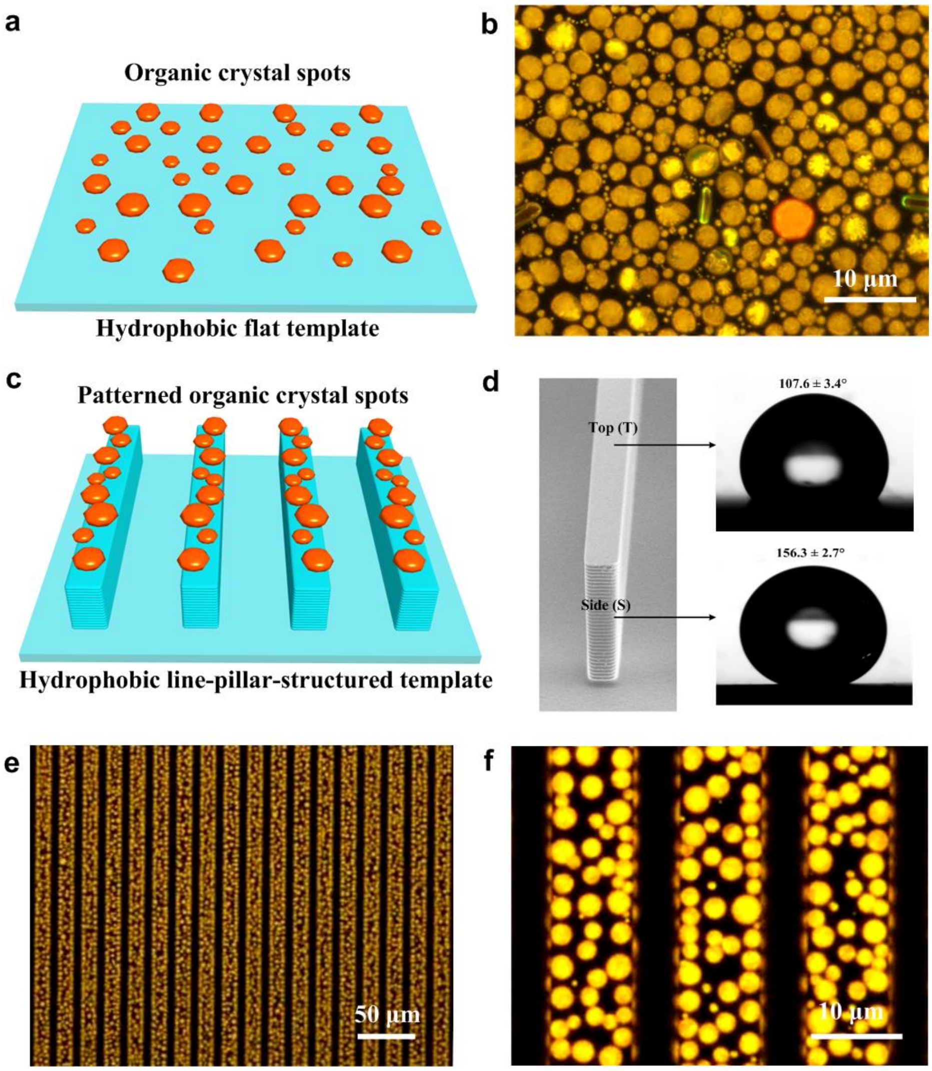

Supplementary Figure 12 | Low-surface-energy micropillars lead to round BPEA depositions upon pillar tops. Schematic illustrations of BPEA depositions upon (**a**) flat and (**c**) groove-structured silicon substrates modified by heptadecafluorodecyltrimethoxysilane (FAS). Dark-field fluorescent micrographs of BPEA depositions upon FAS modified (**b**) flat and (**e**) groove-structured silicon substrates. (**d**) Water contact-angle measurements on the FAS modified pillar tops and sidewalls. Because of the high roughness combined with the hydrophobic nature of silicon, the pillar sidewall surfaces exhibited contact angles (CA) as high as 156.3 ± 2.7°, whereas the flat top regions exhibited CAs of 107.6 ± 3.4°. Since the surface energy of micropillars was reduced by the modification of FAS, BPEA vapor deposited upon pillar tops and sidewalls with round shapes. (**f**) Magnified image of (**e**) showing rough BPEA deposition upon pillar tops.

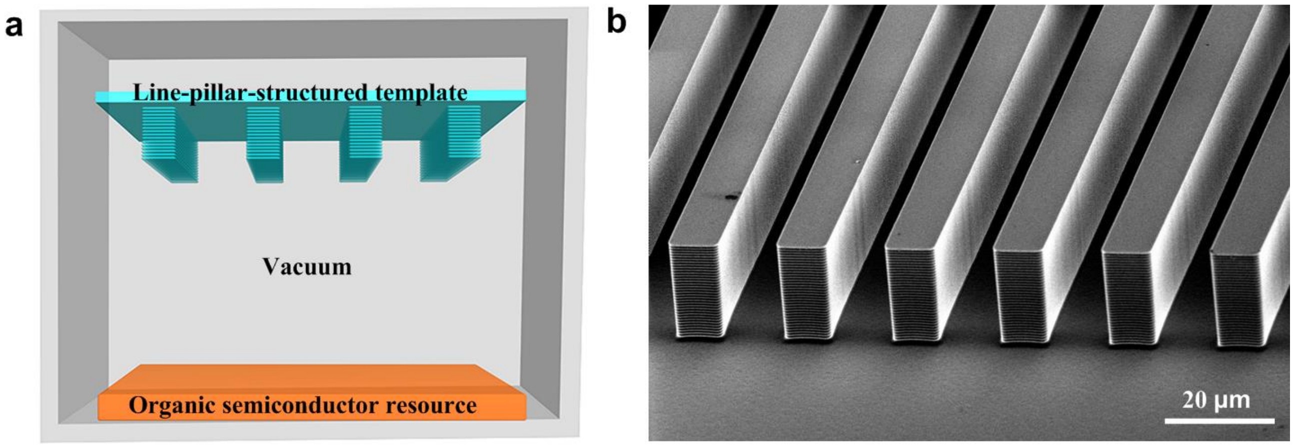

Supplementary Figure 13 | Vacuum environment fail to encourage the growth of BPEA structures. (**a**) Schematic illustrations of the PVT process in a vacuum environment. Owing to the existence of vacuum condition, atmospheric buoyancy has been greatly suppressed. As a result, mass transport from the bottom high temperature zone disappeared, yielding the failure of BPEA crystal growth upon the groove-structured silicon substrate. (**b**) SEM observation of the silicon substrate that has underwent the GPVT process in the vacuum environment. None of BPEA fragments can be found upon the tops or sidewalls of micropillars.

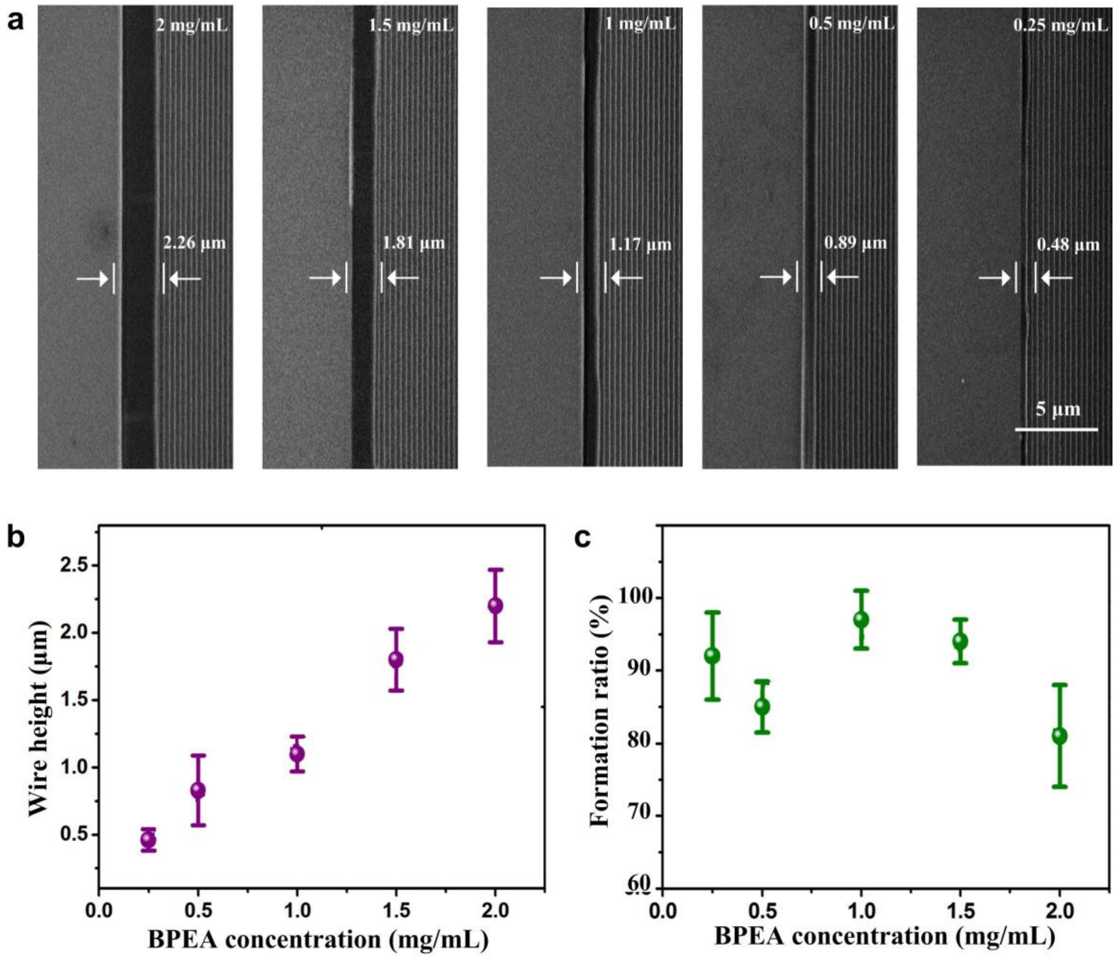

Supplementary Figure 14 | Tunable wire height by reducing the mass transport in the PVGT system. (**a**) Side view SEM observations of aligned BPEA wires with diverse heights depending on the initial concentration. (**b**) The dependence of wire height on the initial concentration. Dilute BPEA liquor indicates limited mass transport during PVGT process. The height of 483 ± 22 nm can thus be generated at the concentration as low as 0.25 mg/mL and increased to several micrometers at 2 mg/mL. (**c**) The dependence of wire formation ratio on the initial concentration. Higher concentration can not benefit the formation ratio of BPEA wires. The data at each concentration have been obtained from 50 samples.

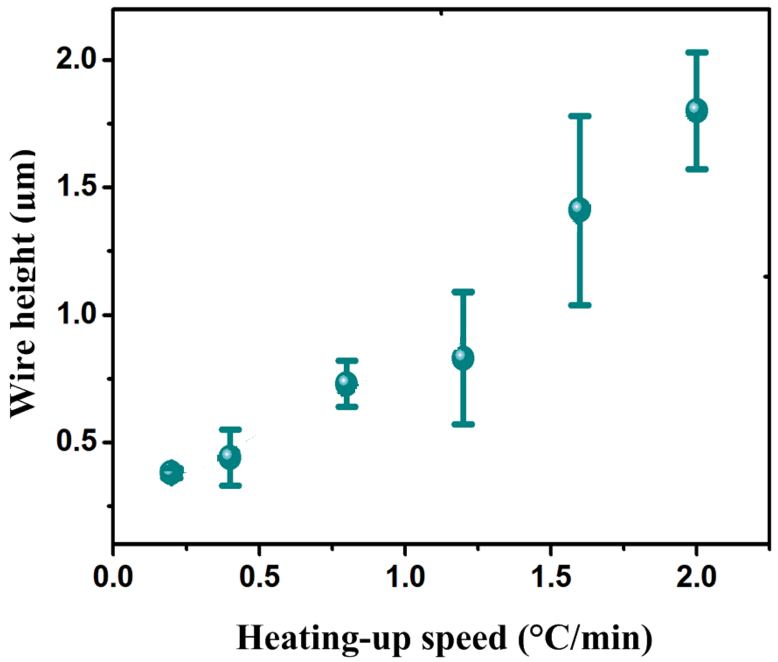

Supplementary Figure 15 | The wire height became larger when increasing the heating-up speed. The dependence of the wire height on the heating-up speed. Higher heating-up speed indicated stronger mass transport supplied from the bottom high temperature zone. Therefore, the height of wires increased accordingly. The data at each heating-up speed have been obtained from 50 samples.

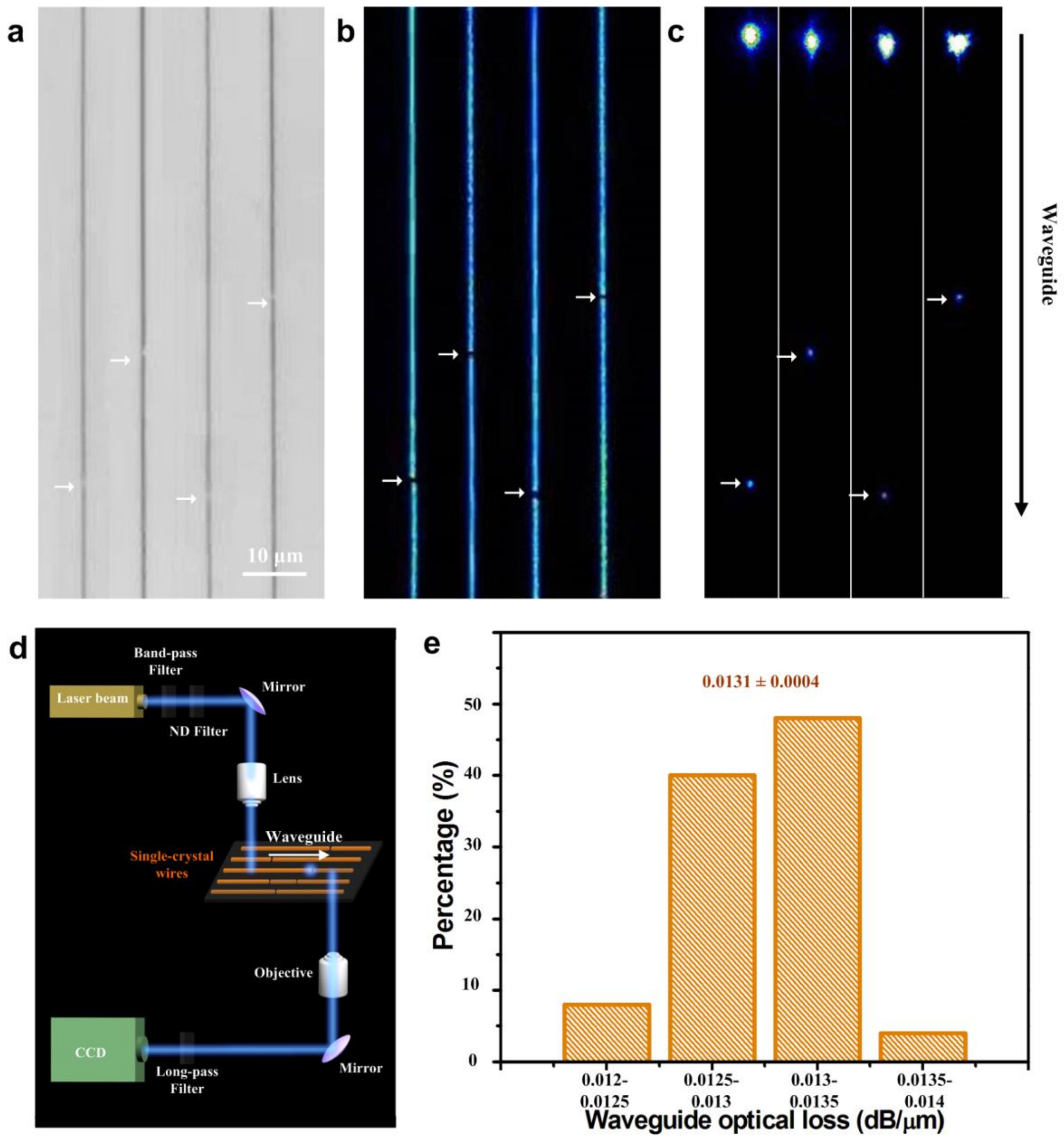

Supplementary Figure 16 | Transferred BPEA wire arrays and their optical waveguide ability. (**a**) Optical and (**b**) in-site fluorescent micrographs of aligned BPEA wires upon a flat polydimethylsiloxane (PDMS) film through a contact transfer technique. Several cleaved points can be found along the wires due to mechanical damage during transfer process, see the white arrows. (**c**) PL images of the waveguiding wire patterns under laser excitation. A 488 nm continuous wave argon-ion laser was focused to a beam spot size of 1 μm to excite aligned organic single-crystal wire at an excitation power of < 2 W/cm^2. Notably, visible luminescence spot appeared at each broken point of the BPEA wires, indicating the focused light propagated along these organic 1D architectures. (**d**) Schematic demonstration of the experimental setup for optical waveguide measurement. The blue lines represent the excitation and observation paths, respectively. The excitation laser (488 nm) was reflected by a dichroic mirror and focused by an objective to excite BPEA wires. The guided PL light was collected by the other objective. Scattered laser light was rejected by a notch filter. **e**, Histograms of the optical loss along the aligned BPEA wires obtained from 50 samples. The average loss is 0.0131 ± 0.0004 dB/μm.

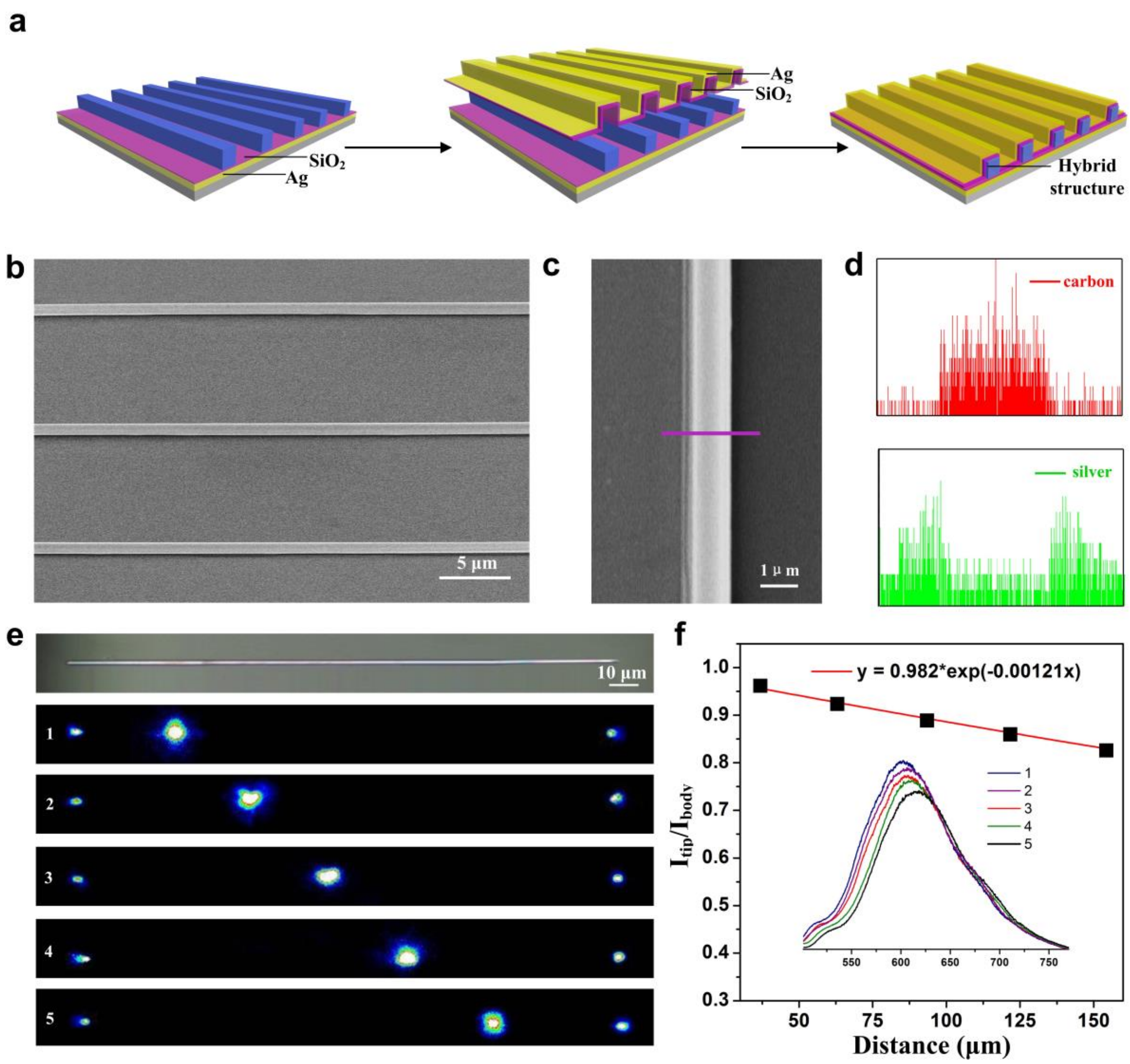

Supplementary Figure S17 | 'Hybrid plasmonics' approach for restraining optical loss. (**a**) Schematic illustration of 'hybrid plasmonics' approach. First, the BPEA wires were fabricated onto the Ag (300 nm)/SiO_2 (5 nm) substrates. SiO_2 (5 nm) and Ag (100nm) film were then deposited on the surface of the BPEA wires to form SiO_2 and Ag encapsulated BPEA wires (BPEA@SiO_2@Ag). SEM images of (**b**) BPEA@SiO_2@Ag wire arrays and (**c**) single wire. (**d**) EDS linear scanning capture from the position marked in **c**. (**e**) Bright-field and PL images obtained from a single BPEA wire coated by SiO_2 (5 nm)/Ag (100 nm) by exciting the wire at different positions. Scale bar is 10 µm. (**f**) The ratio of the intensity I_{tip}/I_{body} against the distance D. Curves were fitted by an exponential decay function $I_{tip}/I_{body} = A\exp(-RD)$. Inset: Spatially resolved PL spectra from the tip of the wire for different separation distances between the excitation spot and tip of the wire shown in **e**.

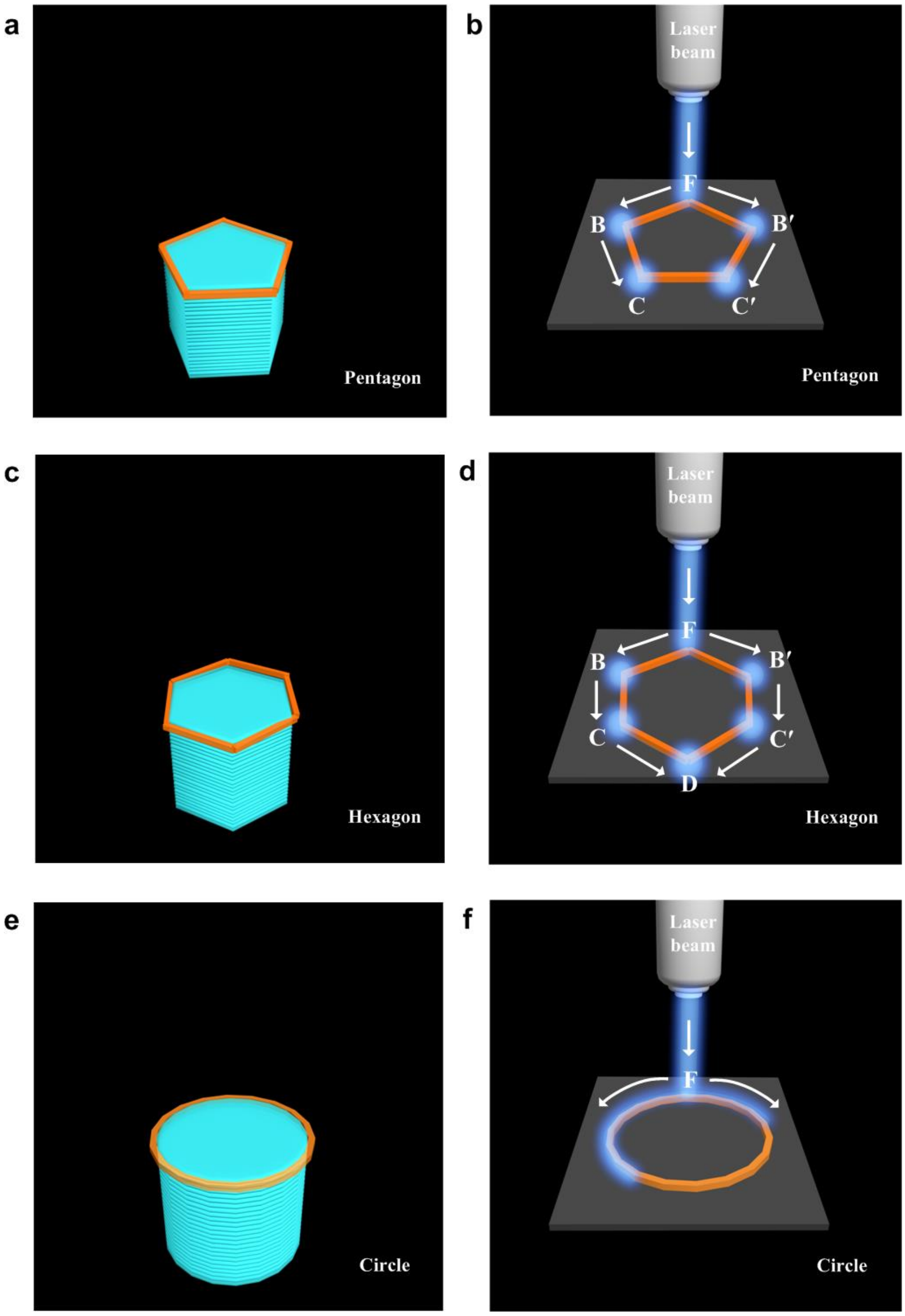

Supplementary Figure 18 | Controlled joining of organic single-crystal wires dominated by the pillar geometry. Since organic wires exhibit edge-growth model in the GPVT process, tailoring pillar geometry, such as (**a**) pentagon, (**c**) hexagon, and (**e**) circle shaped micropillars, will develop joining organic 1D structures within precise included angles and positions. Schematic illustration of an optical waveguide along (**b**) pentagon, (**d**) hexagon, and (**f**) circle patterned organic wires. A 488 nm laser beam was focused onto the wires to investigate the optical propagation.

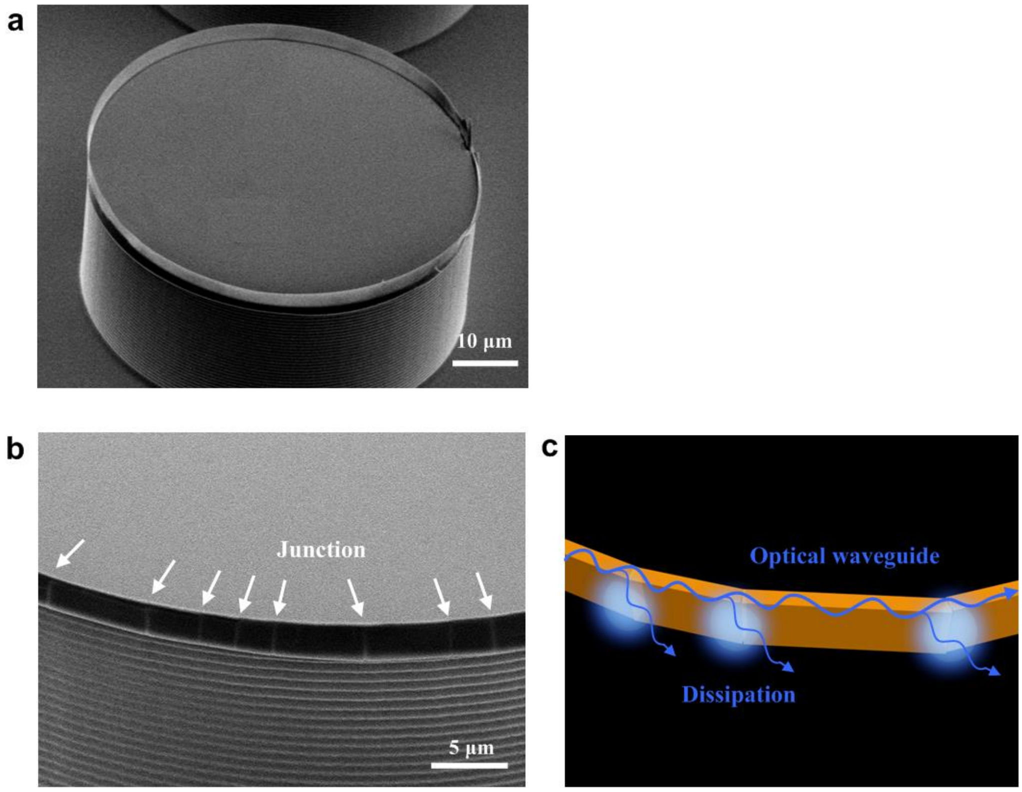

Supplementary Figure 19 | A circle-shaped BPEA wire pattern exhibit reduced propagation distance due to a series of wire joining points. (**a**) A SEM investigation of patterned BPEA wires along a circle shaped micropillar. (**b**) The magnified image of **a** showing considerable joining due to numerous external angles (circle can be regarded as polygon with a plenty of *ca*. 180° external angles). (**c**) Schematic illustration of transport loss at external-angle-dominated joining of wires. As a result, the propagation distance along circle-patterned wires was thus restricted greatly.

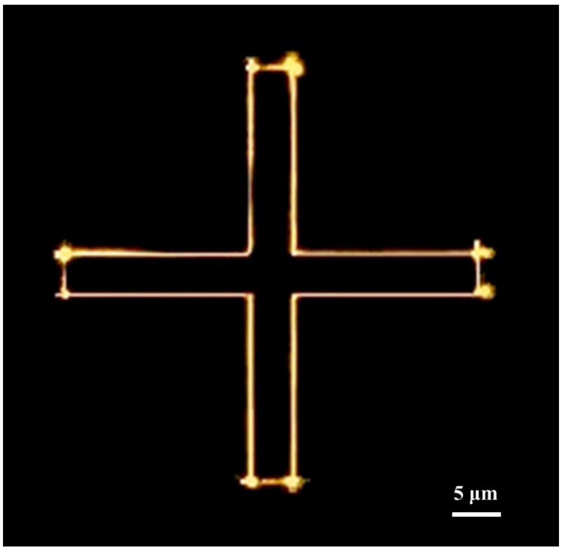

Supplementary Figure 20 | Continuous wire patterns grown along a "X" shaped micropillar through the GPVT process. A "X" shaped micropillar, consisting of both 90° interior angles and external ones, has been utilized to guide the joining of BPEA wires. Dark-field fluorescent micrograph of these patterned BPEA wires shows they are continuous at the 90° joining regions.

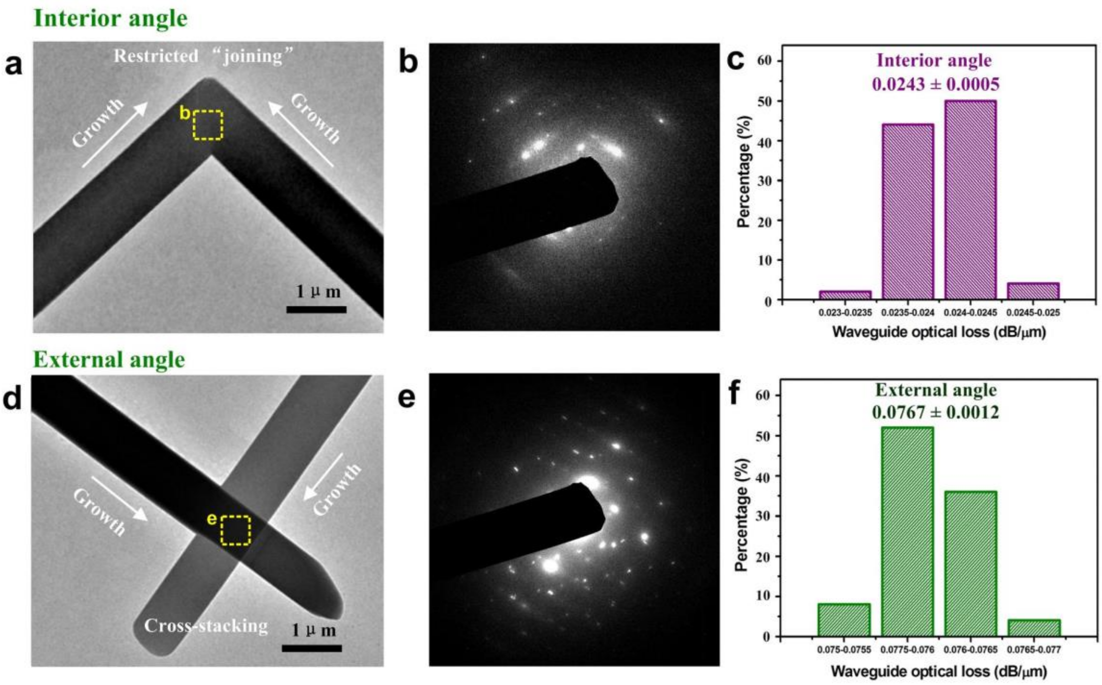

Supplementary Figure 21 | Optical loss caused by interior/external angle mediated joining of wires. TEM observations of 90° joining of wires at (**a**) interior and (**d**) external angle regions. (**b**) and (**e**) are SAED patterns in the wire joining regions of **a** and **d**, respectively. Different joining models of BPEA wires lead to diverse optical loss when driving light transporting along these corner paths. Histograms of the optical loss along the (**c**) interior and (**f**) external angle mediated wire joining obtained from 50 samples. The average loss along interior and external angle mediated wire joining are 0.0243 ± 0.0005 dB/μm and 0.0767 ± 0.0012 dB/μm, respectively.

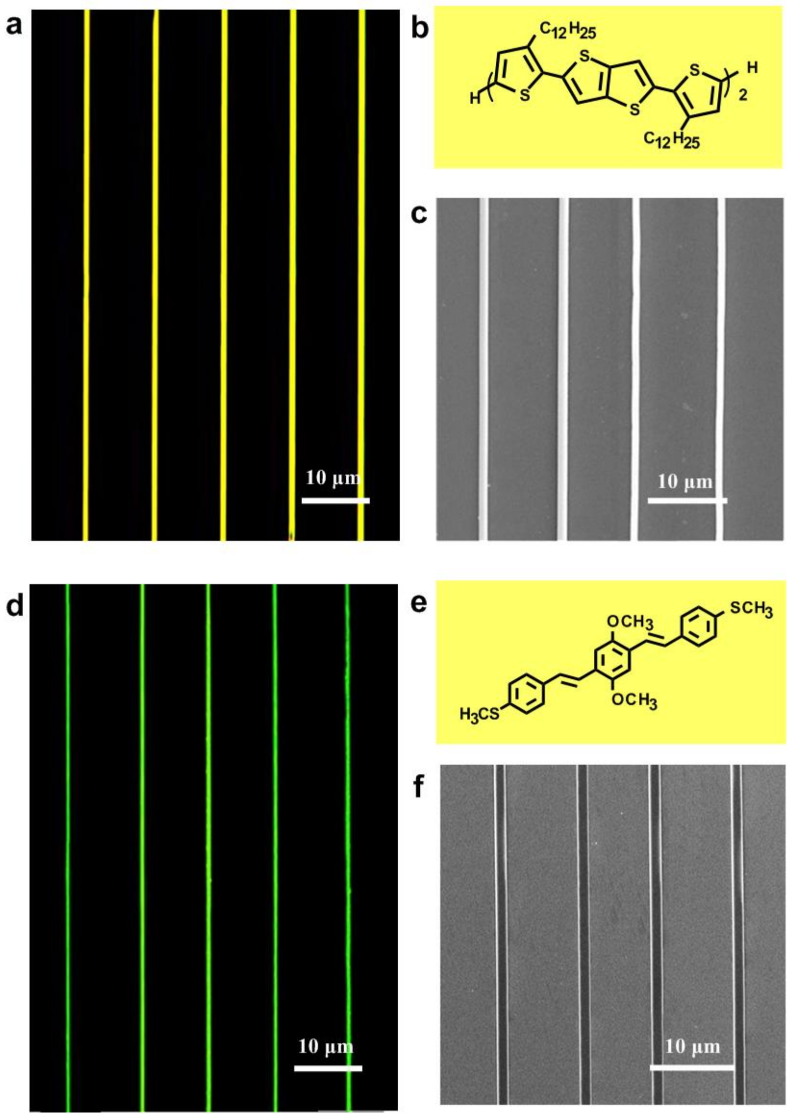

Supplementary Figure 22 | Diverse organic semiconductors can be encouraged to generate highly aligned wire arrays. Dark-field fluorescent micrographs of highly oriented (**a**) di-2,5-bis(dodecylthiophene)-thieno[3,2-b]thiophene (DTTT) and (**d**) 1,4-dimethoxy-2,5-di[4'-(methylthio)styryl]benzene (TDSB) wires upon a flat polydimethylsiloxane (PDMS) film exposed at 325 nm UV irradiation. (**b**, **e**) are molecular structures of DTTT and TDSB, respectively. (**c**, **f**) are SEM observations of aligned DTTT and TDSB wires, respectively.

Supplementary Table 1 | Crystal data and structure refinement for BPEA crystal

**Formula**	**BPEA crystal**
formula weight	378.4
crystal system	monoclinic
space group	C12/c1
a (Å)	22.535(14)
b (Å)	5.356(3)
c (Å)	16.749(11)
α (^{o})	90
β (^{o})	98.678(9)
γ (^{o})	90
V (Å^3)	1998(2)
Z	4
T (K)	173.15
D_c (g cm^{-3})	1.258
F000	792
R_1, ωR_2 [$I > 2\sigma(I)$]	0.0676, 0.1372
R_1, ωR_2 (all data)	0.0748, 0.1411

$^{a}R_1 = \Sigma\ ||F_o| - |F_c||/\Sigma|F_o|$, $\omega R_2 = [\Sigma(|F_o|^2 - |F_c|^2)/\ \Sigma|F_o|^2]^{1/2}$

Supplementary Note 1

Stable BPEA wires grown through the GPVT process in air.

To evaluate the influence of atmosphere (especially oxygen) on the crystal growth, we performed the GPVT process in air at similar experimental conditions, followed by the characterizations of the stability and crystal structure.

Thermogravimetric analysis (TGA), infrared spectroscopy (IR) and ultraviolet (UV) absorption spectrum were performed to evaluate the stability of BPEA grown at the temperature of 200 °C in air. The TGA curve and the corresponding derivative thermogravimetry (DTG) analysis were carried out in air at a heating rate of 5 ^{o}C/min in Supplementary Fig. 7a. These results show that the BPEA start to degrade at about 350 ^{o}C, a temperature much higher than that during the GPVT process (200 °C). In other words, the BPEA molecules were stable without the degradation during the GPVT process. No obvious change of peak position and intensity in the UV absorption spectrum (Supplementary Fig. 7b), indicating the aggregated structure of BPEA molecules are stable after heating at 200 ^{o}C in air. The results from IR spectroscopy also proved this point. Neither a significant band shift nor the appearance of any new band was observed in the IR spectra, which manifested that the molecular structure of BPEA has not been degraded after the heating process (Supplementary Fig. 7c).

On the other side, SEM, TEM, SAED were carried out to investigate the effect of atmosphere on the morphology as well as crystallinity of as-prepared BPEA wires. The SEM and AFM images (Supplementary Fig. 8b-d) indicate that BPEA wires were uniformly aligned with smooth outer surface, which has no obvious difference compared to that grown in nitrogen (Fig.1). The TEM and SAED results (Supplementary Fig. 8e) reveal the single crystallinity of the as-prepared wires, which has the same crystal structure and growth orientation compared to that grown in nitrogen (Fig.1).

Based on those above results, it could be concluded that the BPEA molecules were stable and the wires exhibited single-crystalline structure under 200 ^{o}C through the GPVT process in air.

Supplementary Note 2

Calculation of transport loss in optical waveguide along organic straight wires

Under the irradiation of a focused laser (λ = 488 nm), the BPEA emitted intense broad-band PL. A part of the energy at the exciting spot was scattered into free space while the residual was confined and propagated along the wire. Supposing that the ratio of the light escaping from the excitation point and that of light propagating along the fiber is η, and the collection chance of total scattering by an objective is k, the confined PL intensity focused onto the wire (I_{ex}) can be expressed as:

$$I_{\mathrm{ex}} = \frac{I_{\mathrm{in}}}{k\eta} \qquad (1)$$

where I_{in} is PL intensity of the focused laser beam.

During the propagation of light, the energy loss is contributed by two fractions. The former one is the optical loss (α) along the increasing of propagation. The intensity of the tip emission decreases exponentially with the propagation distance. The latter one is caused by the broken points, which exist during the wire transfer or immature growth. These energy losses (I_n) could be represented directly by the intensity of the luminescence spots. Therefore, the propagation distance of the wires could be expressed as:

$$\mathrm{L} = \frac{1}{\alpha}\ln\frac{(I_{\mathrm{in}}/k\eta - \Sigma_{j=1}^{n} I_{\mathrm{n}}/k\eta)}{I_{\mathrm{tip}}/k\eta} \qquad (2)$$

Where n represents the number of broken points, I_{tip} denotes the PL intensity at the final point. The PL intensity is recorded from corresponding spectra. After a certain mathematic treatment, the equation would be simplified as:

$$\mathrm{L} = \frac{1}{\alpha}\ln\frac{I_{\mathrm{in}} - nI_{\mathrm{def}}}{I_{\mathrm{tip}}} \qquad (3)$$

According to the equation (3), the transport loss in **Figure 1h** in manuscript can be calculated as 0.0131 ± 0.0004 dB/μm, which is a little higher than inorganic wire counterpart[3] yet smaller than that of nanoparticle linear assemblies[4].

Supplementary Note 3

Restraining optical loss by 'hybrid plasmonics' approach

The loss in organic wires is due to the relatively low phase refraction index ($n_r \approx 1.8$) of BPEA nature[3]. Therefore, we introduce the 'hybrid plasmonics' approach to fabricate the plasmonic BPEA wires to eliminate this loss of propagation of the organic materials[5]. Firstly, the Ag (300 nm)/SiO_2 (5 nm) substrates were deposited. And then the BPEA wires were fabricated onto the Ag (300 nm)/ SiO_2 (5 nm) substrates. SiO_2 (5 nm) and Ag (100 nm) film were deposited on the surface of the BPEA wires. The configuration of SiO_2 and Ag encapsulated BPEA wires (BPEA@SiO_2@Ag) is shown in Supplementary Fig. 17a. To characterize the surficial state and the structure of BPEA@SiO_2@Ag, SEM observation and energy dispersive spectroscopy (EDS) linear scanning were performed. SEM image show a relatively smooth surface, which indicates that SiO_2 and Ag film are homogenously deposited (Supplementary Fig. 17b). Further element distribution analysis by EDS linear scanning shows that Ag signal is stronger in margin of wires and C signal mainly exist in the area of core (Supplementary Fig. 17c), which indicates that the BPEA wires are encapsulated by Ag film.

To evaluate the optical loss after the 'hybrid plasmonics' treatment, spatially resolved PL imaging and spectroscopy measurements were performed by locally exciting a single BPEA@SiO_2@Ag wire with a 488 nm focused laser beam. The micro-area PL images obtained from a wire (length l = 205 μm) by accurately shifting the excitation laser spots are shown in Supplementary Fig. 17e. In this one-dimensional (1D) structure, the formation of the surface plasmonics polaritons (SPP) are effectively confined in this 1D active media and propagate along the axis of the wires. The propagation loss of the wire was evaluated by looking at the spatially resolved spectra of the emitted light with respect to the distance travelled. The bottom inset of Supplementary Fig. 17f illustrates the corresponding PL signals detected from the wire terminus by changing the position of the excitation laser beam. The distance dependent intensity of the wire, shown in Supplementary Fig. 17f, indicates that the intensity of the out-coupled light decays almost exponentially with the increase in propagation distance, which is a typical characteristic of active waveguides. The intensity at the excited site along the body of the wire (I_{body}) and at the emitting tip (I_{tip}) were recorded and the optical-loss coefficient (R) was calculated by single-exponential fitting $I_{tip}/I_{body} = A\exp(-RD)$, where D is the distance between the excited site and the emitting tip.

Accordingly, R = 0.00121 dB μm^{-1} at 600 nm, which is much lower than the value for other fabricated pure organic optical waveguide. There are two prominent factors that contribute to the excellent optical waveguide behavior of the BPEA@SiO_2@Ag wires. First, the smooth surface and high crystallinity minimized the optical loss caused by scattering. Second, the formation of surface plasmonics polarition (SPP) can largely reduces the optical loss during the propagation along the length direction of the wire.

Supplementary Note 4

Calculation of transport loss in waveguides along joined wire patterns

According to the description in Note 2 above, the transport loss along the straight wires can be calculated as:

$$\mathrm{L} = \frac{1}{\alpha}\ln\frac{I_{\mathrm{in}} - nI_{\mathrm{def}}}{I_{\mathrm{tip}}} \qquad (3)$$

In order to calculate the optical loss caused by the joining of wires, I_{before} and I_{after} are defined as the PL intensity of the spots that appeared before and after the joining point of wires. In most case, the I_{before} can be regarded as the PL intensity of the focused laser beam.

In this case, the equation (3) can be modified as:

$$L_{total} = \frac{1}{\alpha}\cdot\ln\frac{I_{before}}{I_{after}} \qquad (4)$$

Where L_{total} indicates the added lengths of wires that constitute the joining regions.

Supplementary References

1. Baskaran, A. & Smereka, P. Mechanisms of Stranski-Krastanov growth. *J. Appl. Phys.* **111**, 044321-044327 (2012).

2. Ma, R.-M., Ota, S., Li, Y., Yang, S. & Zhang, X. Explosives detection in a lasing plasmon nanocavity. *Nat Nano* **9**, 600-704 (2014).

3.Solis, D. et al. Electromagnetic Energy Transport in Nanoparticle Chains via Dark Plasmon Modes. *Nano Lett* **12**, 1349-1353 (2012).

4.Law, M. et al. Nanoribbon waveguides for subwavelength photonics integration. *Science* **305**, 1269-1273 (2004).

5. Oulton, R. F. *et al.* Plasmon lasers at deep subwavelength scale. *Nature* **461**, 629-632 (2009).
